# HIV immunological non-responders are characterized by extensive immunosenescence and impaired lymphocyte cytokine production capacity

**DOI:** 10.3389/fimmu.2024.1350065

**Published:** 2024-05-08

**Authors:** Wilhelm A. J. W. Vos, Adriana Navas, Elise M. G. Meeder, Marc J. T. Blaauw, Albert L. Groenendijk, Louise E. van Eekeren, Twan Otten, Nadira Vadaq, Vasiliki Matzaraki, Bram van Cranenbroek, Kees Brinkman, Jan van Lunzen, Leo A. B. Joosten, Mihai G. Netea, Willem L. Blok, Andre J. A. M. van der Ven, Hans J. P. M. Koenen, Janneke E. Stalenhoef

**Affiliations:** ^1^ Department of Internal Medicine, Radboud University Medical Center, Nijmegen, Netherlands; ^2^ Department of Internal Medicine and Infectious Diseases, OLVG, Amsterdam, Netherlands; ^3^ Department of Psychiatry, Radboudumc, Radboud University, Nijmegen, Netherlands; ^4^ Cognition and Behavior, Donders Institute for Brain, Radboud University, Nijmegen, Netherlands; ^5^ Department of Internal Medicine and Infectious Diseases, Elizabeth-Tweesteden Ziekenhuis, Tilburg, Netherlands; ^6^ Department of Internal Medicine, ErasmusMC, Erasmus University, Rotterdam, Netherlands; ^7^ Department of Medical Microbiology and Infectious diseases, ErasmusMC, Erasmus University, Rotterdam, Netherlands; ^8^ Department of Laboratory Medicine, Laboratory for Medical Immunology, Radboud University Medical Center, Nijmegen, Netherlands; ^9^ Department of Medical Genetics, Iuliu Hatieganu University of Medicine and Pharmacy, Cluj-Napoca, Romania; ^10^ Department of Immunology and Metabolism, Life and Medical Sciences Institute, University of Bonn, Bonn, Germany

**Keywords:** immunological non-responders, immune restoration, cytokine production, flow cytometry, immunosenescence, PD1

## Abstract

**Introduction:**

Immunological non-responders (INR) are people living with HIV (PLHIV) who fail to fully restore CD4+ T-cell counts despite complete viral suppression with antiretroviral therapy (ART). INR are at higher risk for non-HIV related morbidity and mortality. Previous research suggest persistent qualitative defects.

**Methods:**

The 2000HIV study (clinical trials NTC03994835) enrolled 1895 PLHIV, divided in a discovery and validation cohort. PLHIV with CD4 T-cell count <350 cells/mm^3^ after ≥2 years of suppressive ART were defined as INR and were compared to immunological responders (IR) with CD4 T-cell count >500 cells/mm^3^. Logistic and rank based regression were used to analyze clinical data, extensive innate and adaptive immunophenotyping, and *ex vivo* monocyte and lymphocyte cytokine production after stimulation with various stimuli.

**Results:**

The discovery cohort consisted of 62 INR and 1224 IR, the validation cohort of 26 INR and 243 IR. INR were older, had more advanced HIV disease before starting ART and had more frequently a history of non-AIDS related malignancy. INR had lower absolute CD4+ T-cell numbers in all subsets. Activated (HLA-DR+, CD38+) and exhausted (PD1+) subpopulations were proportionally increased in CD4 T-cells. Monocyte and granulocyte immunophenotypes were comparable. INR lymphocytes produced less IL-22, IFN-γ, IL-10 and IL-17 to stimuli. In contrast, monocyte cytokine production did not differ. The proportions of CD4+CD38+HLA-DR+ and CD4+PD1+ subpopulations showed an inversed correlation to lymphocyte cytokine production.

**Conclusions:**

INR compared to IR have hyperactivated and exhausted CD4+ T-cells in combination with lymphocyte functional impairment, while innate immune responses were comparable. Our data provide a rationale to consider the use of anti-PD1 therapy in INR.

## 1 Introduction

The prognosis of people living with HIV (PLHIV) has fundamentally improved since the introduction of combination antiretroviral therapy (cART) (1). In general, cART induces viral suppression, increases CD4+ T-cell count (CD4 count), improves immune system functionality, prevents opportunistic diseases and greatly improves life expectancy. A CD4 count restoration to levels ≥500 cells/mm^3^ has even been associated with normal life expectancy in PLHIV (2). Nonetheless, some PLHIV on cART fail to restore their CD4 count to these desired levels despite adequate viral suppression. Collectively this group is known as immunological non-responders (INR), in contrast to immunological responders (IR) who are able to achieve adequate CD4 count restoration during suppressive cART. Being an INR increases the risk of morbidity and mortality, with a higher incidence of non-AIDS malignancies and cardiovascular disease irrespective of viral loads (3–5). More recently, a low CD4 count has been linked to an impaired response to COVID-19 vaccination (6). Older age, delayed initiation of cART and viral co-infections are known risk factors for PLHIV to become an INR (7–10). However, the underlying immunological mechanisms are not fully understood yet.

In the literature there is currently no consensus on the definition of INR (11). Consequently, the described INR incidence ranges widely from 9% - 45% of PLHIV on virologically effective cART (11). The most commonly used definition of INR is a total CD4 count ≤350 cells/mm^3^ after at least two years of virally suppressive therapy (11). Several underlying mechanisms may contribute to the failure to restore CD4 counts to normal levels. Impaired stem cell and thymic output, increased programmed cell death, lymphoid tissue fibrosis, persisting intestinal microbial translocation and augmented inflammation have all been considered (7, 10, 12). Some of these mechanisms may not only affect the phenotype of the circulating immune cells, but also their function. However, in-depth characterization of immune function in INR PLHIV has not been extensively studied before. The suggestion that qualitative immune function may indeed be impaired in INR is fueled by studies where administration of the T-cell proliferative interleukin (IL)-2 did increase absolute CD4+ T-cell counts in INR, but failed to improve clinical endpoints (13).

In the present study, comprehensive immunophenotyping and functional characterization of immune responses was performed in circulating innate and adaptive immune cells comparing INR and IR in two cohorts of virally suppressed PLHIV. Furthermore, the clinical risk factors for INR were analyzed. Such insights could help designing interventions for INR that may not only increase the CD4-cell count but also ameliorate CD4-cell function and thereby improve clinical outcomes of INR.

## 2 Materials and methods

### 2.1 Study population and data collection

The 2000HIV cohort (clinical trials NTC03994835) is a multi-center cohort of 1895 PLHIV enrolled in the Netherlands from 2019-2021.

Baseline characteristics and data collection procedures have been described in detail elsewhere (14). In brief, inclusion criteria were HIV-1 positivity, ≥18 years of age, ≥6 months on cART and latest viral load <200 copies/ml. Exclusion criteria were pregnancy or signs of active infection. Blood samples were collected in EDTA tubes after ≥4 hours of fasting, shipped overnight and processed the next morning.

Participants taking immunomodulatory drugs (methotrexate, prednisone, interleukin inhibitors; n=20) and HIV elite controllers not using cART (n=27) were excluded from this analysis.

In line with the literature, we defined INR based on their most recent CD4 count measurement during regular hospital check-ups. INR were defined as CD4 count ≤350 cells/mm^3^ after at least two years of suppressive cART (11). IR were participants with the most recent CD4 count ≥500 cells/mm^3^, as this level is associated with normal life expectancy (2). Participants who did not match either of these two definitions were excluded from the analysis.

### 2.2 Ethics

The 2000HIV study protocol (clinicaltrials NTC03994835) was approved by the accredited medical research ethics committee Nijmegen (NL68056.091.81). Informed consent was obtained from all participants. The principles of the Declaration of Helsinki were followed.

### 2.3 Hemocytometry

Hemocytometric procedures have been published previously (14). We analyzed whole blood using the Sysmex XN series hematology analyzer (Sysmex, Kobe, Japan) for absolute and relative immune cell counts (15).

### 2.4 Flow cytometry

Blood samples were immunophenotyped using three flow cytometry panels and processed using a twenty-one color, six-laser CytoFLEX-LX (Beckman Coulter), as described previously (16). We performed daily quality control and standardization using CytoFLEX Daily QC Fluorospheres (Beckman Coulter, Catalog # B53230), CytoFLEX Daily IR QC Fluorospheres beads (Beckman Coulter, Catalog # C06147) and SPHEROtm Rainbow calibration particles 6-peak (Spherotech Inc, Catalog # RCP-30-5A-6). Data acquisition was performed using CytExpert software 2.3 (Beckman Coulter) and data analysis using a conventional gating strategy with Kaluza V 2.1.2 software. Specific antibodies were selected for identification of 355 populations of the main innate, T- and B-cell subsets. Markers such as HLA-DR, CD38, PD1, PDL-1, CD40, CD307d, CD81 were evaluated to identify perturbations in activation, exhaustion, maturation status and communication in B-, T- cells and innate immune cells. Antibody selection has been described previously in detail (16).

### 2.5 Peripheral blood mononuclear cells (PBMC) cytokine production capacity *in vitro*

*Ex-vivo* PBMC cytokine production capacity was measured by stimulating PBMCs with a variety of stimuli as described previously (14). To measure monocyte production capacity, PBMCs were stimulated using Poly I:C (a toll-like receptor (TLR) 3 agonist), lipopolysaccharide (LPS), imiquimod (a TLR 7 agonist), IL-1α, HIV-Envelope protein (HIV-Env), cytomegalovirus (CMV) protein, and *Streptococcus pneumoniae* (*S. pneumoniae*). After 24 hours we measured production of IL-1 receptor antagonist (IL-1Ra), IL-10, IL-6, IL-8, IL-1β, tumor necrosis factor (TNF), Monocyte Chemoattractant Protein-1 (MCP-1), Macrophage Inflammatory Proteins 1α (MIP-1α) in the supernatant.

In addition, to assess lymphocyte production capacity, we stimulated PBMCs with *Candida albicans* conidia (*C. albicans* con), *Candida albicans hyphae* (*C. albicans* hyphae), *Escherichia coli* (*E. coli*), *Mycobacterium tuberculosis* (MTB), phytohaemagglutinin (PHA), *Staphylococcus aureus* (*S. aureus*) and *S. pneumoniae*. After 7 days we measured IL-22, IL-5, IL-10, interferon-γ (IFN-γ), and IL-17. After stimulation, supernatant was collected and stored at -20°C until measurement of cytokines with enzyme-linked immunosorbent assay (ELISA). Concentrations, manufacturer and catalogue numbers of stimuli can be found elsewhere (14).

### 2.6 Statistics

#### 2.6.1 Analysis of differences between INR and IR

The 2000HIV study consists of two different cohorts: a discovery cohort and an independent validation cohort. The reasoning behind utilizing these two cohorts is that this allows us to use a larger discovery cohort to search for differences between INR and IR, and subsequently use a second smaller cohort to validate any of our findings. Since our measurements are extensive, we corrected for multiple testing in the discovery cohort. In the validation cohort only hypotheses generated by findings from the discovery cohort were tested, and correction for multiple testing was not required. This structure reduces the chances of untrue coincidental findings. An extensive description of the discovery and validation cohort has been published previously (14). Discovery cohort participants were recruited at the HIV outpatient clinic of Radboudumc Nijmegen, OLVG Amsterdam and Erasmus MC Rotterdam, while participants in the validation cohort were recruited at HIV outpatient clinic of a separate large general hospital (Elisabeth-TweeSteden Ziekenhuis Tilburg). Although the samples of the two cohorts were collected separately, processing and measurements were identical.

#### 2.6.2 Clinical data, HIV-related parameters, and comorbidities analysis

We analyzed HIV-related parameters and comorbidities using a logistic regression model with age and sex as covariates. First, we employed the INR/IR group as the dependent variable to identify potential risk factors for INR in clinical data and HIV related parameters. Next, we utilized the INR/IR group as the dependent variable to determine what comorbidities were linked to the INR group. The outcomes of our analysis were presented in the form of odds ratios along with their corresponding 95% confidence intervals.

#### 2.6.3 Complete blood count with differential measurement analysis

Hemocytometric measurements were compared between INR and IR using a Wilcoxon rank sum test, with statistical significance set at p < 0.05.

#### 2.6.4 Flow cytometry data analysis

For absolute cell populations, percentages, and mean fluorescence intensity (MFI) measured by flow cytometry data, we employed a linear regression model comparing INR to IR corrected for relevant confounders. Prior to analysis, flow cytometry data were transformed using inverse rank-based transformation to achieve a normal data distribution. To identify potential confounders, we assessed associations between the first five principal components (PCs) of the flow cytometry data and potential confounders using a linear regression model in the discovery cohort. The results were presented as adjusted R^2^ values, with variables having high adjusted R^2^ values considered as potential confounders (**Supplementary Figures 1A, B**). Final confounder selection was based on changes in beta coefficients exceeding 10% in the linear regression model. This process identified age, sex, seasonality, and COVID-19 vaccination as significant confounders in the discovery cohort. The list of confounders identified in the discovery cohort was applied to the validation cohort.

#### 2.6.5 PBMC cytokine production capacity analysis

Prior to analysis, PBMC cytokine production capacity data were transformed using inverse rank-based transformation to standardize the unit of measurement. Due to non-normal data distribution after transformation, differences between INR and IR in PBMC cytokine production capacity were analyzed using a non-parametric rank-based regression model. We used a Wilcoxon score function for rank-based fitting. This model was corrected for age, sex, and the lymphocyte to monocyte ratio, as measured by the Sysmex hematology analyzer. Correction for lymphocyte to monocyte ratio was used to correct for the difference in PBMC content between INR and IR. As per definition INR have a lower number of lymphocytes and thus a different lymphocyte to monocyte ratio. When a set number of PBMCs is used, INR samples contain a lower number of lymphocytes which could influence the number of cytokines produced. A similar approach for confounder assessment, as used for the flow cytometry data, was applied to the PBMC cytokine production capacity data (**Supplementary Figures 1C, D**). Cytokines produced after 24 hours of stimulation were considered to be monocyte produced cytokine, whereas cytokines produced after 7 days of stimulation were considered to be the result of lymphocyte cytokine production, as previously described and published (17, 18).

#### 2.6.6 Correlation of flow cytometry data and PBMC cytokine production

Data from all participants from the 2000HIV study were used to correlate flow cytometry and PBMC cytokine results. Elite controllers and participants on immunomodulatory drugs remained excluded. Prior to analysis, flow cytometry and PBMC cytokine production results were both transformed using inverse rank-based transformation. Analysis was done using a linear model with confounders as previously observed in the flow cytometry (seasonality, COVID-19 vaccination) and PBMC cytokine production (lymphocyte/monocyte ratio) analyses, and sex and age.

#### 2.6.7 Statistical significance

For flow cytometry, PBMC cytokine production capacity and the correlation between these two analyses, results were considered significant when the P-value false discovery rate (FDR) was less than 0.05 in the discovery cohort, and the nominal p-value (p) was less than 0.05 in the validation cohort. Significant results from the discovery cohort were re-tested in the validation cohort for both significance and similar trend (same directionality of change as observed in the discovery cohort) as measures of validation. All analyses were conducted using R Studio version 4.2.2 (2022-10-31).

## 3 Results

### 3.1 Clinical associations

**Figure 1:**
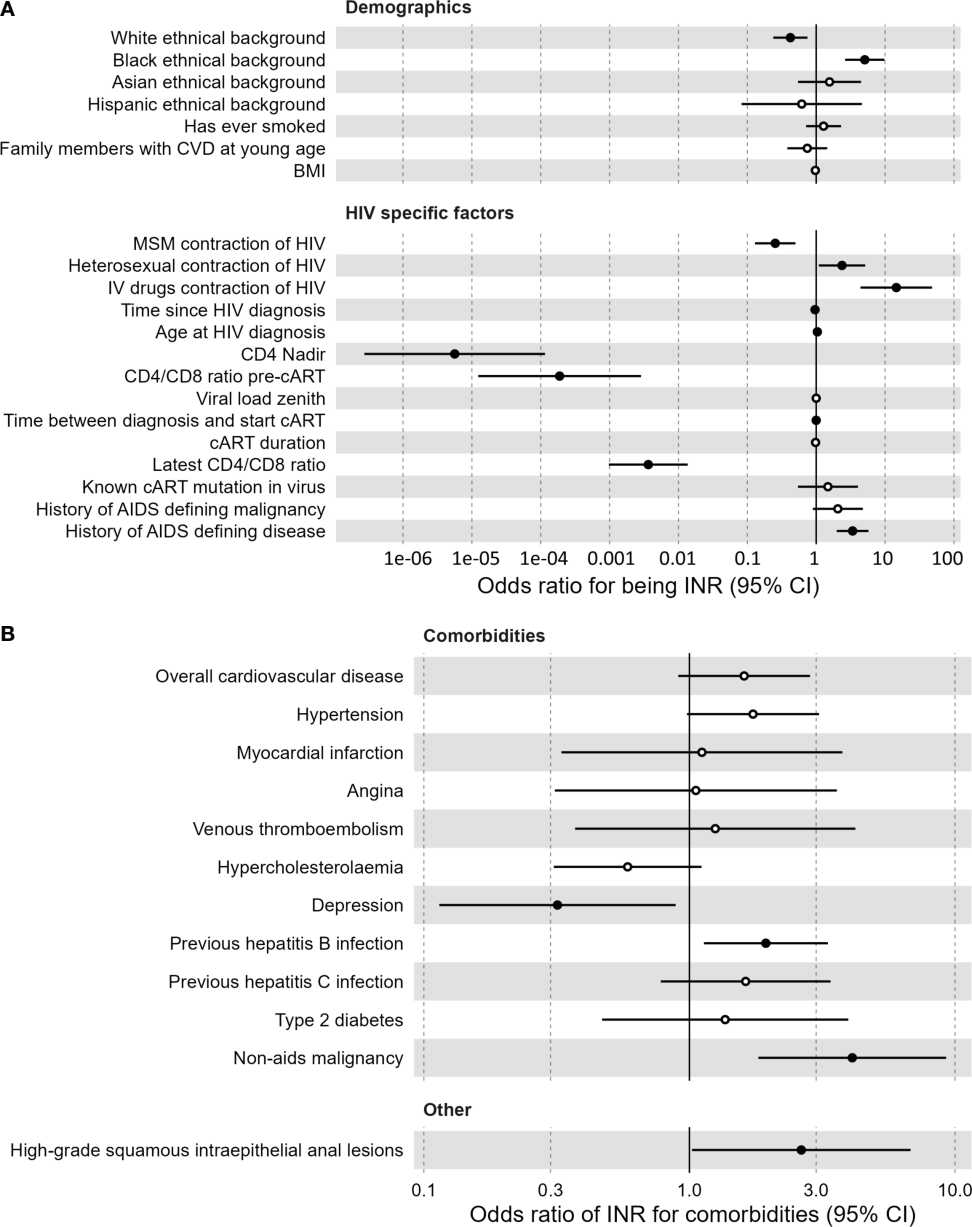
Clinical associations of immunological non-responders compared to immunological responders. **(A)** Demographic and HIV specific factors predictive of the immunological non-responder phenotype, compared to immunological responders. Multivariate logistic model with sex and age as covariates. Black dots indicate a significant difference. Vertical lines indicate confidence interval (CI) of the odds intervals per clinical measurement. Time between diagnosis and start combination antiretroviral therapy is negatively correlated with immunological non-responders. **(B)** Comorbidities for which the immunological non-responder phenotype serves as a predictor, compared to immunological responders. Multivariate logistic model with sex and age as covariates. Black dots indicate a significant difference. Vertical lines indicate confidence interval (CI) of the odds intervals per clinical measurement. BMI, body mass index; cART, combination antiretroviral therapy; CI, confidence interval; CVD, cardiovascular disease; IV, intravenous; MSM, men who have sex with men.

Our study consists of 88 INR and 1476 IR, separated into a discovery cohort (62 INR, 1224 IR) and a validation cohort (26 INR, 243 IR). Baseline characteristics are outlined in **Supplementary Table 1**. In an univariate logistic model applied to the discovery cohort, older age was predictive of INR (odds ratio (OR)=1.025; p=0.032) but sex assigned at birth (sex) was not (**Supplementary Figures 2A, B**). To generalize associations, corrections for sex and age were made in all subsequent analyses. Multivariate logistic regression revealed multiple significant predictors of INR status in the discovery cohort (**Figure 1A**; **Supplementary Figures 2C, E**). Black ethnicity (OR=5.08, p<0.001), older age at initial HIV diagnosis (OR=1.040, p=0.035), HIV transmission through intravenous drug use (OR=14.6, p<0.001) and heterosexually acquired HIV infection (OR=2.37, p=0.029) were positively correlated with being an INR. Also, factors indicative of advanced HIV disease pre-cART such as low CD4 nadir (OR<0.001, p<0.001), low CD4/CD8 ratio pre-cART (OR<0.001, p<0.001), and a history of AIDS defining disease (OR=2.1, p<0.001) were associated with INR. INR were also associated with lower most recently measured CD4/CD8 ratio (OR=0.014, p<0.001). Finally, INR are known to have increased risk for non-AIDS comorbidities. Using a multivariate logistic model with sex and age as covariates, we investigated if being an INR was predictive of comorbidities in the discovery cohort (**Figure 1B**). We found that INR were more likely to have a history of a hepatitis B infection (anti-HBc+, OR=1.94, p=0.015) and a history of a non-AIDS defining malignancy (OR=4.10, p<0.001). No association was observed between INR and cardiovascular disease (p=0.1).

Of the HIV specific factors mentioned above, only the lower CD4 nadir of the INR could be significantly validated in the validation cohort, possibly due to the limited sample size compared to the discovery cohort (**Supplementary Figures 2D, F**). Finally, current cART regimen was not associated with the INR phenotype (**Supplementary Figures 2G, H**, **Supplementary Tables 2G, 2H)**. An extensive list of all clinical associations for both cohorts can be found in **Supplementary Figure 2** and **Supplementary Table 2**.

### 3.2 Hemocytometry

**Figure 2:**
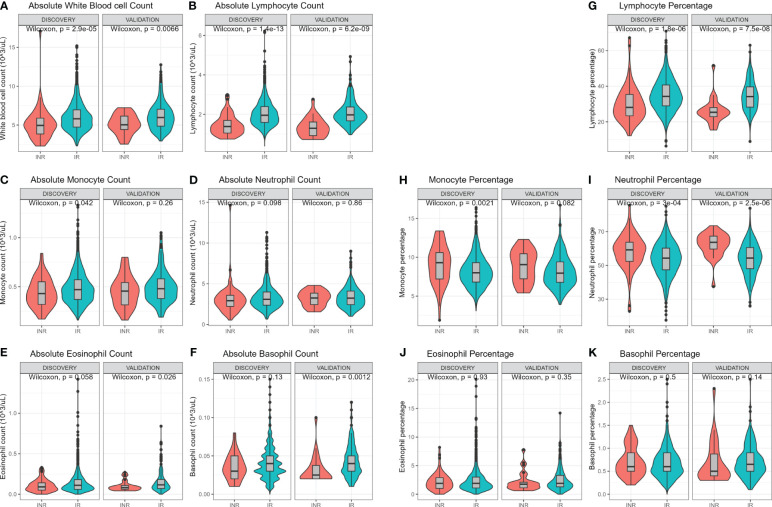
Hemocytometric comparison of immunological non-responders and immunological responders. Comparisons through Wilcoxon rank sum test. Comparison of immune cell counts in immunological non-responders compared to immunological responders: Full white blood cell count **(A)**, lymphocyte count **(B)**, monocyte count **(C)**, neutrophil count **(D)**, eosinophil count **(E)**, and basophil count **(F)**. Immune cells relative percentages as part of white blood count in immunological non-responders compared to immunological responders: Lymphocyte percentage **(G)**, monocyte percentage **(H)**, neutrophil percentage **(I)**, eosinophil percentage **(J)**, and basophil percentage **(K)**. INR, immunological non-responders; IR, immunological responders.

White blood cell (WBC) count and differentiation were measured using a hematology analyzer (**Figure 2**). Immune cell subsets can be expressed as absolute counts or as percentage of WBC. In both cohorts INR had lower absolute WBC counts (discovery median 5.0 vs 5.8 x10^3^ cells/µL, p=<0.001; validation median 5.0 vs 6.0 x10^3^ cells/µL, p=0.007; **Figure 2A**) and lower absolute lymphocyte counts (discovery median 1.4 vs 2.0 x10^3^ cells/µL, p<0.001, validation median 1.3 vs 2.0 x10^3^ cells/µL, p<0.001; **Figure 2B**). INR had lower absolute monocyte count in the discovery cohort (p=0.042) but not in the validation cohort (p=0.26) (**Figure 2C**). Absolute counts of neutrophils, eosinophils and basophils did not differ (**Figures 2D–F**). Furthermore, the percentage of the various immune cells in relation to WBC count were investigated. The percentage of lymphocytes was lower in INR compared to IR in both the discovery (median 28% vs 34%, p<0.001) and validation cohort (median 25.4% vs 34.2%, p<0.001) (**Figure 2G**). The percentage of monocytes was higher in INR in the discovery cohort (median 9.3% vs 8%, p=0.002) and showed a similar trend in the validation cohort (median 9.1% vs 8.0%, p=0.082) (**Figure 2H**). Neutrophil percentage was significantly higher in INR in the discovery (median 59.2% vs 54.3%, p<0.001) and validation cohort (63.7% vs 54.4%, p<0.001) (**Figure 2I**). Eosinophil and basophil percentages did not differ (**Figures 2J, K**). Overall, this shows that WBC count and WBC composition are different in INR compared to IR.

### 3.3 Immunophenotyping

Circulating immune cells were characterized in depth by flow cytometry. Immune cell characterization of all 355 subsets can be found in **Supplementary Figure 3**. Principal component (PC) analysis showed that seasonality and past COVID-19 vaccination influenced the absolute and proportional flow cytometry data (**Supplementary Figures 1A, B**). A correction for these factors was made in addition to sex and age when comparing a broad spectrum of innate and adaptive immune cells.

**Figure 3:**
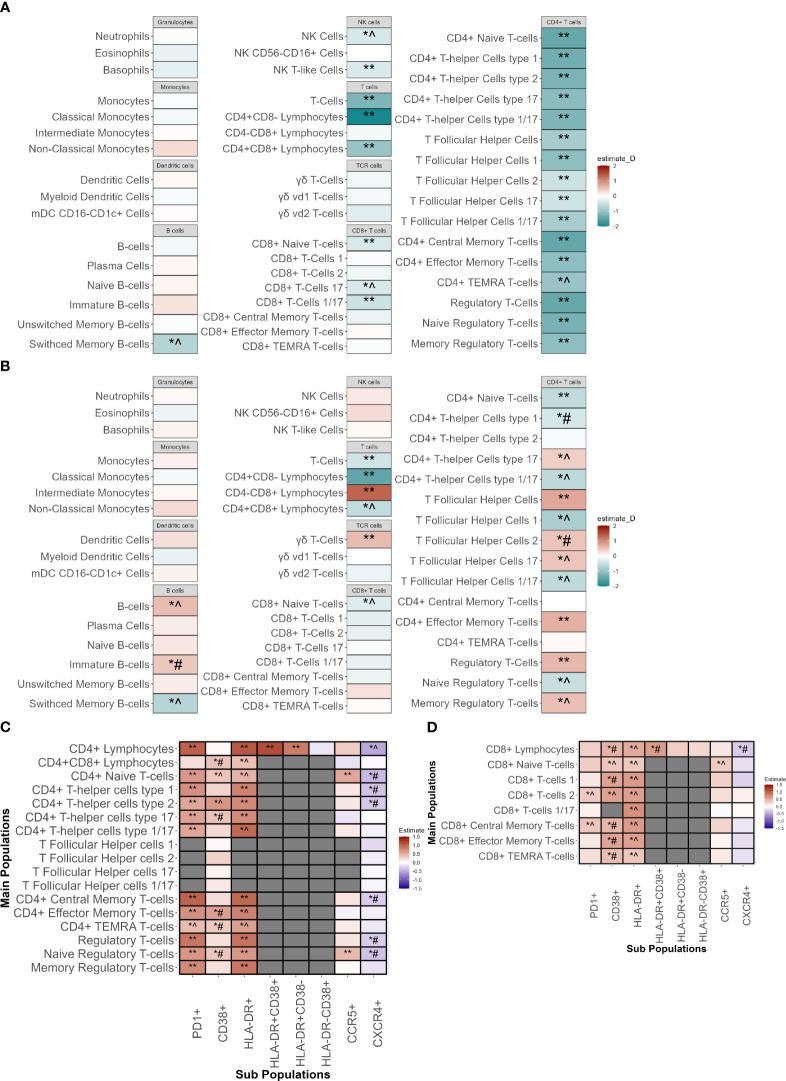
Immunophenotyping of innate and adaptive immune cells of immunological non-responders compared to immunological responders. Comparison through a linear model using sex, age, seasonality and COVID-19 vaccination as covariates. ** Indicates significant difference between immunological non-responders and immunological responders in both the discovery and validation cohort with equal directionality of the estimate. *^ indicates significance in the discovery cohort and equal directionality change of the estimate in the validation cohort without significance. *# indicates significance in the discovery cohort but with contradictory directionality change in validation cohort. **(A)** Absolute counts of immune cell types in immunological non-responders compared to immunological responders of the major innate and adaptive immune cell populations with estimates from the discovery cohort. **(B)** Percentages of immune cell types in immunological non-responders compared to immunological responders of the major innate and adaptive immune cell populations with estimates from the discovery cohort. Estimates shown are from the discovery cohort. **(C)** Percentages of CD4+ T-cell subpopulations in immunological non-responders compared to immunological responders in the discovery and validation cohort. Y-axis shows the main cell populations, X-axis displays the receptor that is added to the main population to depict the subpopulation. Estimates shown are from the discovery cohort. **(D)** Percentages of CD8+ T-cell subpopulations in immunological non-responders compared to immunological responders in the discovery and validation cohort. Y-axis shows the main cell populations, X-axis displays the receptor that is added to the main population to depict the subpopulation. Estimates shown are from the discovery cohort. mDC, Myeloid dendritic cells; NK-cells, natural killer cells; TEMRA, T effector memory cells re-expressing CD45RA.

First, the absolute cell counts were compared between INR and IR (**Figure 3A**; **Supplementary Figure 4A**). In the discovery cohort INR had lower absolute CD4+ T-cells counts including all CD4+ subpopulations, of which most could be significantly validated. In INR, CD8+ T-cells were reduced in the CD8+ Naive T-cells (CD8_Naive_), and in the CD8+ T-cell 17 (CD8_TC17_) and CD8+ T-cell 1/17 (CD8_TC1/17_) subsets. In B-cell subsets, the absolute number of switched memory B-cells was lower in INR, a finding that had the same trend in the validation cohort. In innate immunity cells, natural killer (NK) and NK T-like cell numbers were lower in INR. Other innate immune cell counts were similar in INR and IR.

Subsequently, the relative proportions of immune cells in INR and IR were compared to elucidate differences in the distribution of cells and relative immune changes (**Figure 3B**; **Supplementary Figure 4B**). In both cohorts, INR had a decreased proportion of CD4+ naive T-cells (CD4_Naive_) and an increased proportion of CD4+ effector memory cells (CD4_TEM_). Percentages of CD4+ T-follicular helper cells (CD4_Tfh_) and regulatory T-cells (T_reg_) were significantly increased in INR in both cohorts. In the CD4_Tfh_ subsets of the discovery cohort we observed an increase of the CD4_Tfh17_ and CD4_Tfh2_ fractions and a decrease in the CD4_Tfh1_ and CD4_Tfh1/17_ fractions. These findings showed a similar trend in the validation cohort but did not reach significance, except for the CD4_Tfh2_ subpopulation that showed an opposite trend. In addition, within the CD4+ T-helper cell (CD4_Th_) subsets we saw a similar tendency with a proportional increase in CD4_Th17_ but a decrease in CD4_Th1/17_, with similar non-significant trends in the validation cohort. We observed no changes in CD4_Th2_. Concerning CD8+ T-cells, we found a significantly validated increased percentage of CD8+CD4- cells alongside with a decreased percentage of CD8_Naive_ in the validation cohort that showed an equal trend in the validation cohort. Also, an increase in the fraction of γδ T-cells and a decrease in switched memory B-cells (SMBC) was observed in INR. Lastly, no differences were observed in overall percentages of granulocytes, monocytes, dendritic cells or NK-cells.

Next, we assessed subsets for markers of activation and exhaustion. In line with our findings above, the absolute counts of almost all CD4+ and some CD8+ and NK subpopulations were decreased in INR (**Supplementary Figure 4A**). We evaluated the percentages of subsets expressing functional markers for activation and exhaustion. The frequencies of all CD4 T-cell subsets positive for HLA-DR, CD38 (indicative of activation) and PD1 (associated with activation and exhaustion), were significantly higher in the discovery cohort (**Figure 3C**). These findings were significantly validated in the validation cohort, except for the increase of CD38+ subpopulations.

Regarding CD8+ T-cells, in the discovery cohort we found increased percentages of HLA-DR+ and CD38+ subpopulations, but only a limited increase in PD1+ subpopulations (**Figure 3D**). The increase in HLA-DR+ and PD1+ subpopulations showed a similar non-significant trend in the validation cohort whereas the CD38+ subset showed a trend towards lower in the validation cohort.

Regarding the innate immune cells, we observed that INR had increases in the HLA-DR+ subpopulations of NK cells and NK T-like cells (similar trends in the validation cohort) (**Supplementary Figure 4C**). In addition, classical monocytes showed an increase in percentage of PDL1+ subsets with an opposite trend in the validation cohort (**Supplementary Figure 4D**). Comparisons of all population measurements with their absolute counts (**Supplementary Figure 4A**; **Supplementary Tables 3A**, **3B**) and percentages (**Supplementary Figure 4B**; **Supplementary Tables 3C**, **3D**) can be found in the **Supplementary Figures and Tables**.

Finally, since the main differences in the CD4+ T-cell compartment were observed in HLA-DR, CD38 and PD1 subsets, we compared the signal intensity of these markers per cell (mean fluorescence intensity (MFI)) between INR and IR in all CD4+ cell subsets, after correcting for sex, age, COVID-19 vaccination status and seasonality (**Supplementary Figure 4E**). In the discovery cohort the CD4+ T-cell populations of INR showed an increased MFI of PD1 (16/17 populations) and CD38 (14/17 populations). PD1 MFI showed a (significant) similar trend in the validation cohort in 14/16 populations, and CD38 in 13/14 populations. Upregulation of HLA-DR MFI was less pronounced in INR. This shows that INR do not only have increased percentages of cells expressing PD1 on CD4+ T-cells, but also increased PD1 expression per single cell.

Increased T-cell PD1+ expression is well known in cancers and a broadly used target in cancer therapies (19). To exclude the possibility that the observed increase in PD1+ subpopulations in INR was caused due to the heightened prevalence of past malignancies observed in INR, we repeated our analysis but excluded all participants with a history of cancer. Flow cytometry results were unchanged (data not shown). This shows that our finding of increased CD4+PD1+ subpopulations in INR is independent of the higher incidence of history of cancers observed in INR.

Some IR definitions state that IR should be defined as PLHIV who also had a low CD4 nadir (CD4 <350 cells/mm^3^) and subsequently then were able to restore their CD4 count > 500 cells/mm^3^. Therefore, we repeated the analyses with IR who all had CD4 nadir <350 cells/mm^3^. This did not influence the observed results (data not shown).

Overall, analyzing the flow cytometric data, major alterations in lymphocyte subsets were observed, with increased proportions of activated and exhausted subpopulation in the CD4+ subsets of INR.

### 3.4 PBMC *ex vivo* cytokine production capacity

Increased proportions of HLA-DR+ and PD1+ cells are markers of cellular activation and exhaustion. To investigate whether this translated into impaired immune cell functionality, we stimulated PBMC with several stimuli *ex vivo* and measured cytokine production capacity after 24 hours and after 7 days to measure monocyte and lymphocyte cytokine production capacity, respectively. As WBC content ratios differed between INR and IR as described above, we corrected for lymphocyte/monocyte ratio as measured by hemocytometry as well as for sex and age.

**Figure 4:**
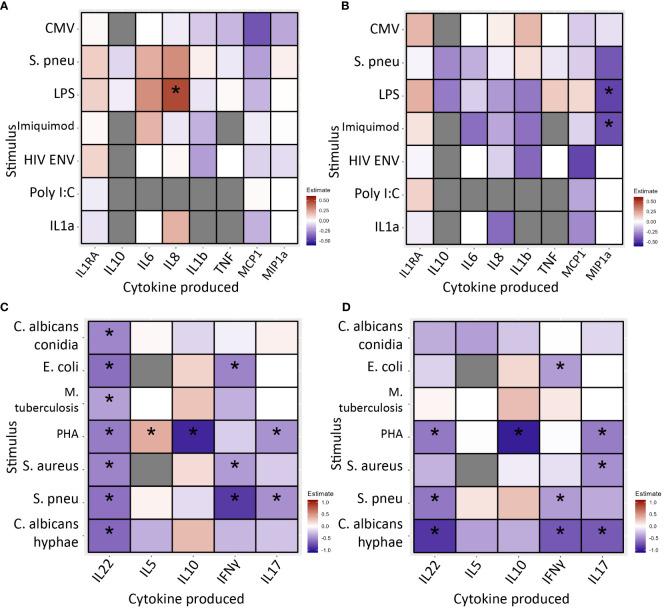
PBMC cytokine production in immunological non-responders compared to immunological responders after 24 hours **(A, B)** and 7 days **(C, D)** of stimulation with various stimuli, * indicates significance. Stimuli are shown on the Y-axis, produced cytokine on the X-axis. Blue/light blue indicates decreased cytokine production in INR compared to IR, red/orange indicates increased. Black stars specify statistical significant difference (<0.05) after FDR correction in the discovery cohort and nominal p-value in the validation cohort. Analysis through linear regression using sex, age and lymphocyte/monocyte ratio as covariates. **(A)** In the discovery cohort after 24 hours of stimulation immunological non-responders showed comparable cytokine production in response to most stimuli. Immunological non-responders only showed increased IL-8 production to LPS. **(B)** In the validation cohort after 24 hours of stimulation immunological non-responders showed comparable cytokine production in response to most stimuli. The significant finding from the discovery cohort could not be validated. **(C)** In the discovery cohort after 7 days of stimulation immunological non-responders showed reduced cytokine production in response to several stimuli. IL-22 production was significantly reduced in response to six out of seven stimuli, IL-10 was decreased after PHA stimulation, IFNγ production was lower in response to *E.coli*, MTB, *S. aureus* and *S. pneumoniae* stimulation. IL-17 production was decreased after PHA and *S. pneumoniae* stimulation. **(D)** In the validation cohort seven observed decreases of cytokine production could be significantly validated. All other significant findings from the discovery cohort showed a similar trend. PBMC, peripheral blood mononuclear cells; CMV, Cytomegalovirus protein; HIVENV, HIV envelope protein; IL1RA: Interleukin 1 receptor antagonist; IL, Interleukin; IMQ, Imiquimod; MCP1, Monocyte Chemoattractant Protein-1; LPS, Lipopolysaccharide; MIP-1α, Macrophage Inflammatory Proteins 1α; PBMC, peripheral blood mononuclear cell; PolyIC, Polyinosinic:polycytidylic acid; Spneu, *Streptococcus pneumoniae.* C. alb.con, *Candida albicans* conidia; C. alb.hy, *Candida albicans* hyphae; E.coli, *Escherichia coli*; IFN, interferon; IL, interleukin; MTB, *Mycobacterium tuberculosis*; PHA, phytohemagglutinin**;** S. aureus, *Staphylococcus aureus*; S. pneu, *Streptococcus pneumoniae*; TNF, tumor necrosis factor.

After 24 hours of stimulation, monocyte cytokine production was similar in INR and IR (**Figures 4A, B**; **Supplementary Tables 4A**, **4B**).

Secondly, PBMCs were stimulated for 7 days to measure lymphocyte cytokine production capacity. Overall, we found decreased cytokine production in INR (**Figures 4C, D**; **Supplementary Tables 4C**, **4D**). Most notably, in the discovery cohort IL-22 production was decreased in response to all stimuli; IL-10 was reduced in response to PHA; IFN-γ was decreased after stimulation with *E. coli*, *S. aureus* and *S. pneumoniae*; and IL-17 responses were lower upon PHA and *S. pneumoniae* stimulation. The only increase was seen in IL-5 production in response to PHA. In the validation cohort, we significantly validated seven of these decreased conditions. Except for IL-22 production in response to MTB, all other findings showed a similar trend. IL-5 production in response to PHA was marginally increased and with similar trend in the validation cohort (estimate=0.009, p=0.969).

Overall, in INR cytokine production capacity appears unchanged in monocytes but decreased in lymphocytes indicating reduced functional responsiveness of lymphocytes.

### 3.5 Correlation of immune activation and exhaustion with cytokine production

Lastly, we correlated the proportion of CD4+ T-cell subpopulations indicating immune activation (CD4+CD38+HLA-DR+) and exhaustion (CD4+PD1+) with lymphocyte cytokine production capacity after seven-day stimulation. In this analysis we used all the participants in the discovery and validation cohorts, including participants that could not be categorized as INR or IR. We hypothesized a correlation independent of IR/INR phenotyping and tested this in all participants of the 2000HIV study. Elite controllers without cART and participants using immunomodulatory drugs were excluded. Data were corrected for seasonality, COVID-19 vaccination status, lymphocyte/monocyte ratio, age and sex. In the discovery cohort, the proportion of CD4+CD38+HLA-DR+ subpopulations was inversely correlated with most IL-22, IL-17, and IFN-γ production (**Supplementary Figure 5A**). Inversed correlations were most notably observed in the conditions that showed reduced cytokine production in INR. The validation cohort did not reach significance but all negative estimates in the discovery cohort were also found negative in the validation cohort, which could indicate a lack of statistical power due to low absolute numbers (**Supplementary Figure 5B**).

Correlations between proportions of CD4+PD1+ subpopulations were present but less abundant. In the discovery cohort we observed two inversed correlations with IL-22 production, one with IL-5 production, three with IFN-γ production and two with IL-17 production (**Supplementary Figure 5C**).

Findings showed similar trends in the validation cohort (**Supplementary Figure 5D**).

Only including INR and IR instead of the full cohorts in this analysis did not change the results (data not shown). Overall, our data indicate that PLHIV with lymphocytes showing signs of activation and/or exhaustion, also show cytokine production hyporesponsiveness.

## 4 Discussion

Immunological non-responders remain a vulnerable group, despite the success of cART in the great majority of PLHIV (5). A comprehensive understanding of their functional immune deficits may help to identify targets to improve their prognosis. Our data show that INR significantly differ in immune phenotypes from IR and that functional differences are mostly found in the lymphocyte subsets, while changes in the innate immune system were limited. More specifically, markers of immune activation and exhaustion were found in all CD4+ T-cell subsets and in some CD8+ and NK subsets in INR. Also, impaired lymphocyte cytokine production capacity was observed after various *ex vivo* stimuli. The CD4+ T-cell markers for immune activation and exhaustion, and lymphocyte cytokine production capacity were inversely correlated. Our results point to a persisting immune dysfunction in INR despite well controlled viremia. Not only do INR have lower CD4 T-cell numbers, but also exhibit a significant increase of exhaustion markers on CD4 T-cells. Moreover, the decrease in lymphocyte-derived cytokine production capacity indicates impaired functionality. This functional immune impairment likely contributes to the morbidity and mortality observed in INR, and these markers of immune activation or exhaustion may possibly identify patients at risk. The notable increased expression of PD1+ on all subsets of CD4 T-cells of INR highlight the potential of immune checkpoint inhibition with PD1 blockers as a possible intervention target.

Older age and delayed initiation of cART were both associated with INR status in our cohorts. This is in line with previous reports from other cohorts (5, 7–10). Hepatitis B and C have been inconsistently described as potential risk factor of immunological non-response (20, 21). However, this association was only found for hepatitis B in our study. Some risk factors for INR cannot be modified, while others, such as the early detection and treatment of HIV-infection and hepatitis B vaccination among risk groups, may reduce the risk of becoming an INR. Possibly the positive association between INR and acquisition of HIV through heterosexual transmission could also in this regard be explained, as HIV testing is less abundant in the heterosexual than the MSM (men who have sex with men) community.

Impaired hematopoiesis and thymus dysfunction have been proposed as important mechanisms underlying insufficient immune restoration in INR (22, 23). Our finding of reduced absolute and proportional CD4_Naive_ and CD8_Naive_ counts in INR is in concordance with this hypothesis. We observed similar trends in CD4_Tfh_ and CD4_Th_ with decreased proportions of type 1 and type 1/17 subsets in INR. Type 1 helper cells are especially important in intracellular immunity, such as immunity against viruses and cancer. A decreased subset of this immunophenotype could indicate hampered intracellular immunity.

Furthermore, increases of CD4+ subsets expressing the activation marker HLA-DR and the exhaustion marker PD1 have been previously described in INR (24–27). Shive et al. showed increased percentages of PD1+ subsets in CD4+ central memory cells (CD4_CM_), CD4+ effector memory cells (CD4_EM_) cells and CD4+ terminal effector memory cells (CD4_TEMRA_), but not in CD4+_naive_ (27). In contrast, Han et al. showed an increased PD1+ subset in CD8 effector memory (CD8_EM_) subpopulations only, but not in other CD8+ or CD4+ subpopulations (28). CD8+ T-cells and NK cells both showed HLA-DR+ subpopulations that were comparable to the situation in CD4+ T-cells, although the PD1+ subpopulation increase was limited. This indicates that exhaustion is less prominent in CD8+ T-cells and NK cells. However, HLA-DR expression on NK cells has been associated with NK-cell cytotoxicity against uninfected CD4+ T-cells (29).

The counts and proportions of CD38+ cells (another well-known activation marker) in CD4+, CD8+ and NK-cell subsets showed no consistent pattern in the discovery and validation cohort. The upregulation of CD38+ cells in HIV patients has been consistently reported, and co-expression of HLA-DR and CD38 has been suggested to mirror a state of immune hyperactivation (30). Our finding that percentages of combined HLA-DR+CD38+ subsets are upregulated in INR on CD4+ T-cells in both cohorts, but are inconsistent in CD8+ T-cells, indicates that hyperactivation in INR is most evident in CD4+ T-cells.

Furthermore, a proportional increase of PD1+ subpopulations in CD4+ subsets was noticed, indicating immune exhaustion in these subsets (26). Previously Cockerham et al. described that MFI of PD1+ on CD4+ T-cells was higher in INR compared to IR and healthy controls, but was similar in IR and healthy controls (31). This is in line with our findings increased PD1 MFI in almost all CD4 subpopulations (**Supplementary Figure 4E**). This could indicate that INR are suffering from an increase in PD1 compared to healthy controls, rather than IR having decreased PD1. We found an increased proportion of PD1+ subpopulations in CD4_naive_ T cells of INR, in contrast with the finding of Shive et al. (25). Our data show that the upregulation of CD4 subset percentages expressing these markers are both widespread and consistent throughout all CD4+ subsets, indicating that all CD4+ T-cell subsets are dysregulated in INR. T-cell exhaustion is part of immunosenescence which is independently associated with increased morbidity and mortality (32). If activated by its ligand, PD1 inhibits T-cell function, which in a healthy state prevents autoimmunity. An increase in PD1 expressing subsets can potentially lead to excessive immune system inhibition, thereby attenuating immune functionality. In these cases, PD1 is known to contribute to T-cell dysfunction, while blocking of inhibitory immune checkpoints may possibly restore T-cell functions in HIV (33). PD1 inhibitors have even been associated with distinct immune changes with increased circulating Tfh responses and antibody response to vaccination (34). Based on our findings, we hypothesize that PD1 inhibitors could potentially improve immune functionality in INR. As far as we know, this potential intervention has not been tested in INR yet. The scientific attention of PD1 in PLHIV has been on improving cytotoxic CD8+ T-cell function and reducing the viral reservoir (35). PD1 inhibitor administration in SIV rhesus macaques showed improved T-cell functionality, faster viral suppression and increased Th_17_ reconstitution in the gut (36). Increased gut permeability and microbial translocation is another potential mechanism for INR phenotype (7, 10). Currently, there are phase 1 and 2 trials ongoing to test the concept of PD1 antagonism in HIV cure (NCT05330143, NCT04223804, NCT04554966). Inclusion criteria of all these trials are, however, a high (at least >300) CD4+ T-cell count. Given our current findings in INR we suggest that PD1 antagonism might be of particular interest for PLHIV that are INR.

Furthermore, our data indicate that INR have similar *ex vivo* cytokine production capacity compared to IR after 24 hours of stimulation, but show decreased cytokine production after 7 days of stimulation. Previous investigations into cytokine production capacity of INR have been done in small groups, with limited stimuli and/or limited cytokine measurements. In these small studies, INR compared to IR produced similar innate immune cell cytokines (37, 38) but lower lymphocyte derived cytokines (39). These results are in line with our findings. In a study by Erikstup et al., including 18 INR cases and 35 IR controls, only 24 hours whole blood PHA and LPS stimulation was done. They showed reduced production of IL-10, TNF, IL-2 and IL-5 to PHA stimulation and reduced production of only IL-10 to endotoxin stimulation in INR (40). No differences were found for IFN-γ, IL-6 or IL-8. Most of these findings are in line with our findings, although we did not find differences in IL-10 production or TNF production after 24h of stimulation. A possible explanation could be that Erikstrup et al. used whole blood samples and that they used Mann Whitney U tests to calculate differences, whereas we used PBMCs and a rank-based model adjusted for sex, age and lymphocyte/monocyte ratio. Especially correcting for lymphocyte/monocyte ratio shows that the decrease in lymphocyte produced cytokines is independent of the ratio of cells present. Particularly the observed decreases in IL-22, IL-10 and IL-17 with a downward trend for IFN-γ in response to PHA are noteworthy as PHA is the strongest stimulus we applied. This demonstrates that when fully urged to empty all stored cytokines, lymphocytes of INR produce significantly less than IR.

Also, the cytokine production capacity in INR was inversely correlated to the level of CD4+CD38+HLA-DR+ expression and to CD4+PD1+ expression. This implies that heavily activated or exhausted CD4+ T-cells function differently. It suggests that in INR exhausted immune cell phenotypes appearance (PD1+) is associated with an impaired immune function as measured by cytokine production. Thus, both quantitative as well as qualitative immune disturbances were detected in INR in our study.

The current study has several strengths and limitations. With 1564 participants in two cohorts, 355 immune cell populations in flow cytometry and 12 cytokines in response to 13 stimuli our study offers the most comprehensive exploration ever performed on immune functions of INR. By performing these analyses in the blood of the same participants, we were able to combine immunophenotyping and functional immunological data. Also, using two distinct cohorts allowed for fast and immediate validation of findings. The fact that not all findings could be significantly validated in the validation cohort might be due to the reduced statistical power in this smaller cohort. Therefore, we also reported similar trend as possible indicator of validation. Finally, our lab has extensive experience with flow cytometry and cytokine production measurements, reducing the possibility of lab artefacts.

However, the current study also has some limitations. Our research shows associations and does not allow causal conclusions. Despite the large size of our cohort, the number of INR we could identify was relatively small and we did not have a reliable way to measure and compare time since HIV infection. There are clinical differences between the discovery and validation as they were independently collected. These differences may account for the fact that not all differences observed between INR and IR were reproducible in the validation cohort and is a possible confounder for result replication. Furthermore, PD1 is a surrogate but not an optimal marker for immunosenescence. More specific immunosenescence T-cell markers such as CD57, Tim-3 or KLRG-1 should therefore be further investigated to prove true immunosenescence in INR (32). We did, however, show that increased PD1+ subpopulations correlate to functional hyporesponsiveness. In addition, we considered cytokines produced after 24 hours to be the result of monocyte cytokine production and after 7 days of stimulation to be produced by lymphocytes, based on previous work (17, 18). These are, however, assumptions as in both cases PBMC’s containing both lymphocytes and monocytes were used. We did not perform intracellular cytokine measurement. Finally, our study participants were primarily enrolled during the COVID-19 pandemic. Immune changes due to pandemic associated lockdowns and COVID-19 vaccination have been detected in our cohort (data under review). Although we checked for most notable confounders such as lockdown and vaccination, we cannot exclude that other yet undiscovered effects of this major worldwide event might have influenced our results.

Our study suggests that INR have immune dysfunctionality that goes beyond just the number of CD4+ T-cells. We found over-expression of activation and exhaustion markers mainly on CD4 T-cells, CD8 T-cells and NK cells. Cytokine production capacity was impaired in lymphocytes derived from INR which was inversely correlated with hyperactivation and exhaustion of immune cells in INR. These functional deficits are in line with the finding of an increased incidence of morbidity and mortality which can be observed in INR despite of well controlled viral replication. Based on our results, we hypothesize that INR could benefit from PD1 checkpoint inhibitor therapy to improve immune functionality.

## Data availability statement

The raw data supporting the conclusions of this article will be made available by the authors upon request, without undue reservation.

## Ethics statement

The studies involving humans were approved by Medical research ethics committee Nijmegen, The Netherlands. The studies were conducted in accordance with the local legislation and institutional requirements. The participants provided their written informed consent to participate in this study.

## Author contributions

WV: Data curation, Formal analysis, Investigation, Methodology, Writing – original draft, Visualization. AN: Formal analysis, Methodology, Writing – review & editing. EM: Formal analysis, Writing – review & editing, Visualization. MB: Data curation, Writing – review & editing, Visualization. AG: Data curation, Writing – review & editing. Lv: Data curation, Writing – review & editing. TO: Formal analysis, Writing – review & editing, Visualization. NV: Formal analysis, Methodology, Writing – review & editing, Visualization. VM: Formal analysis, Methodology, Writing – review & editing. Bv: Conceptualization, Methodology, Supervision, Writing – review & editing. KB: Conceptualization, Supervision, Writing – review & editing. Jv: Writing – review & editing, Conceptualization. LJ: Conceptualization, Supervision, Writing – review & editing. MN: Conceptualization, Supervision, Writing – review & editing. WB: Conceptualization, Supervision, Writing – review & editing. AV: Conceptualization, Supervision, Writing – review & editing. HK: Methodology, Supervision, Writing – review & editing. JS: Conceptualization, Supervision, Writing – review & editing.
