# Supplementary material for: HIV immunological non-responders are characterized by extensive immunosenescence and impaired lymphocyte cytokine production capacity
Source: Front Immunol. 2024 May 8;15:1350065. doi: 10.3389/fimmu.2024.1350065 (PMC11109418; doi:10.3389/fimmu.2024.1350065)
Supplement: Supplementary file 1 [file DataSheet_1.docx]

**Supplementary material**

**Supplementary figure 1:** Principal component analysis of flow cytometry (A,B) and cytokine production analyses (C,D).  **Supplementary figure 1A:** Principal component analysis on potential confounders of flow cytometry absolute counts data. Covid vaccination (COVID_VACC) and seasonality (season_cos and season_sin) appeared to influence flow cytometry results and were therefore corrected for in the analysis.


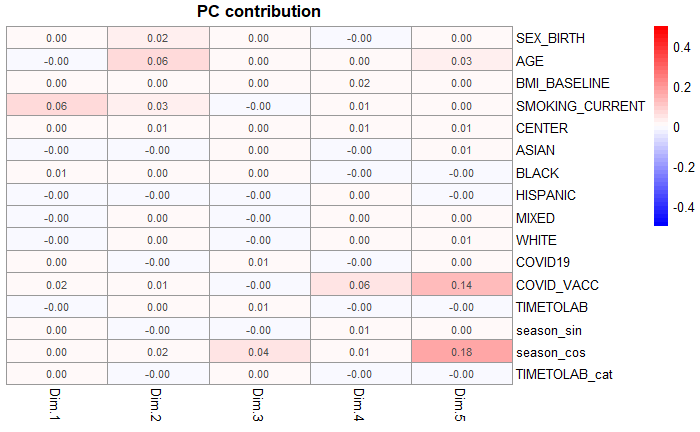


**Supplementary figure 1B:** Principal component analysis on potential confounders of flow cytometry percentage counts data. Covid vaccination (COVID_VACC), seasonality (season_cos and season_sin) and age (AGE) appeared to influence flow cytometry results and were therefore corrected for in the analysis.


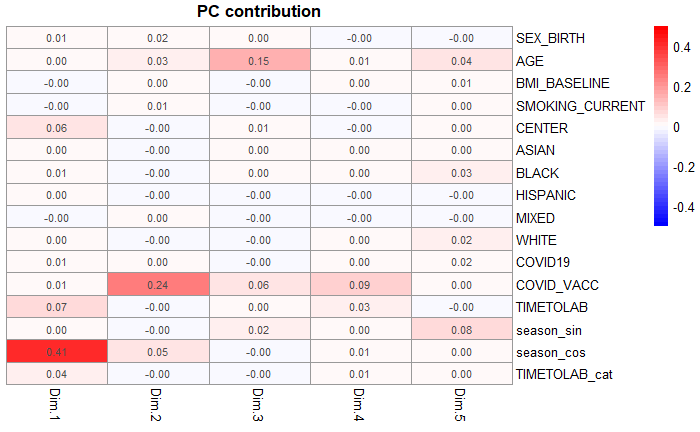


**Supplementary figure 1C:** Principal component analysis on potential confounders of 24h cytokine production data. Several parameters indicative of percentage of monocytes in PBMCs showed to influence the results. We corrected for monocyte to lymphocyte ratio (LYMMONRATIO) as this appeared to most truthfully reflect PBMC content.
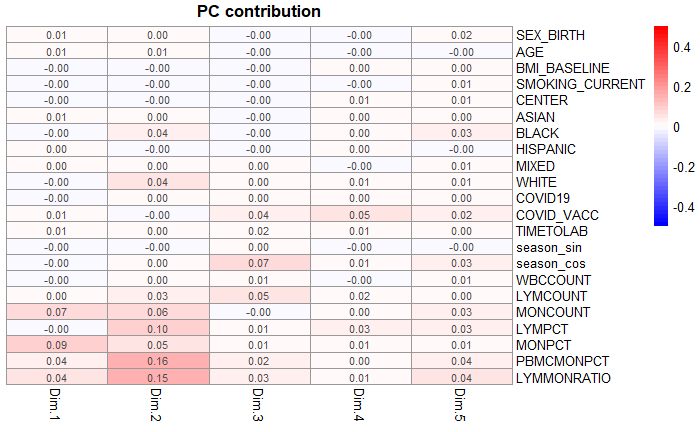


**Supplementary figure 1D:** Principal component analysis on potential confounders of 7d cytokine production data. Several parameters indicative of percentage of lymphocytes of PBMCs showed to influence the results. We corrected for monocyte to lymphocyte ratio (LYMMONRATIO) as this appeared to most truthfully reflect PBMC content.


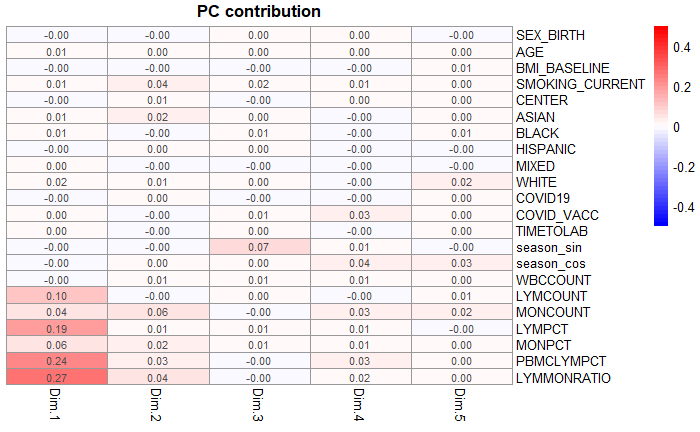


Abbreviations: SEX_BIRTH: sex assigned at birth; BMI: body mass index; CENTER: center where participant was included; COVID_VACC: participant received a COVID-19 vaccination prior to participation; TIMETOLAB: time between sampling and arrival at laboratory; season_cos/season_sin: seasonality; WBCCOUNT: white blood cell count; LYMCOUNT: lymphocyte count; MONCOUNT: monocyte count; LYMPCT: lymphocyte percentage as part of white blood cells; MONPCT monocyte percentage as part of white blood cells; PBMCLYMPCT: lymphocyte percentage as part of peripheral blood mononuclear cells count; LYMMONRATIO: lymphocyte to monocyte ratio. **Supplementary figure 2: Clinical associations of immunological non-responders**
Supplementary figure 2A: Clinical associations of sex and age in relation to immunological non-responders compared to immunological responders in the discovery cohort. Univariate logistic regression analysis. Black dots indicate a significant difference. Vertical lines indicate confidence interval of the odds intervals per clinical measurement. Right of the vertical line indicates positive correlation, left of the vertical line indicates negative correlation.

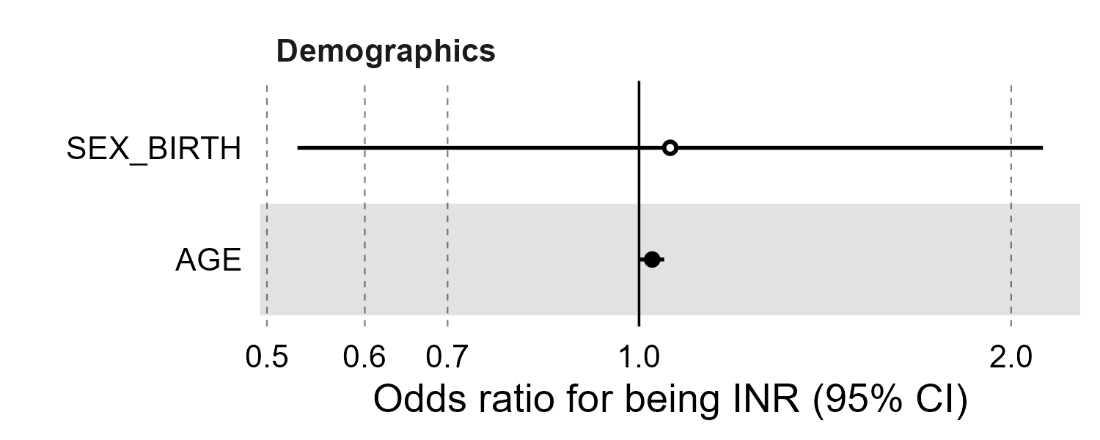


Supplementary figure 2B: Clinical associations of sex and age in relation to immunological non-responders compared to immunological responders in the validation cohort. Univariate logistic regression analysis. Black dots indicate a significant difference. Vertical lines indicate confidence interval of the odds intervals per clinical measurement. Right of the vertical line indicates positive correlation, left of the vertical line indicates negative correlation.


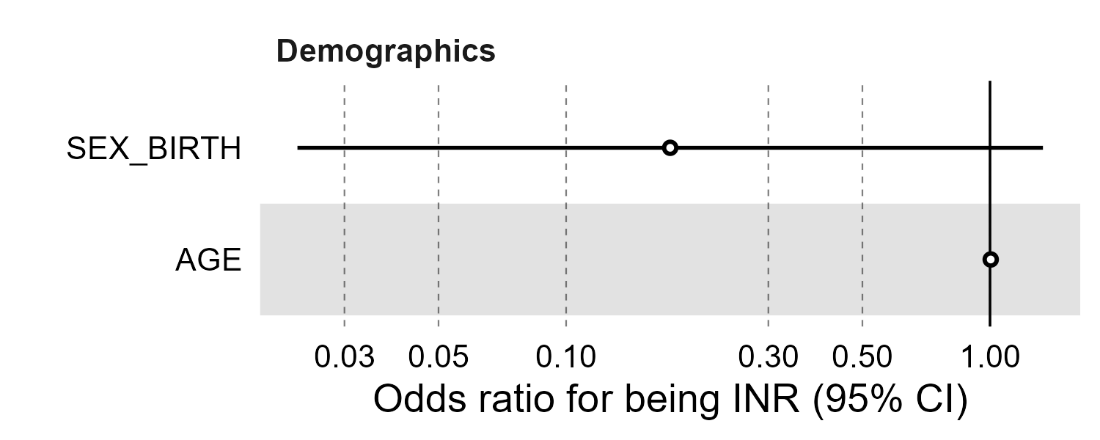


Supplementary figure 2C: Clinical associations of demographic and HIV factors of immunological non-responders compared to immunological responders in the discovery cohort. Multivariate logistic regression analysis with sex and age as covariates.


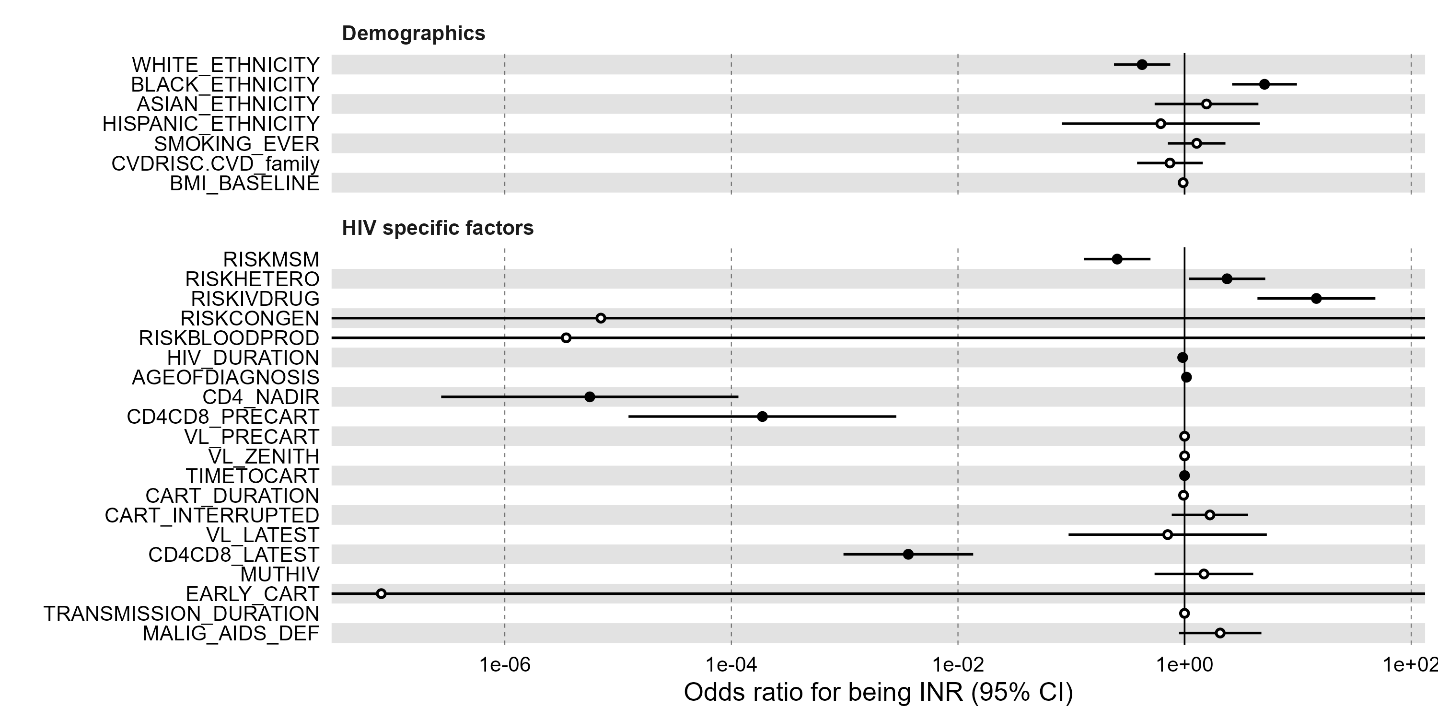


Supplementary figure 2D: Clinical associations of demographic and HIV factors of immunological non-responders compared to immunological responders in the validation cohort. Multivariate logistic regression analysis with sex and age as covariates.


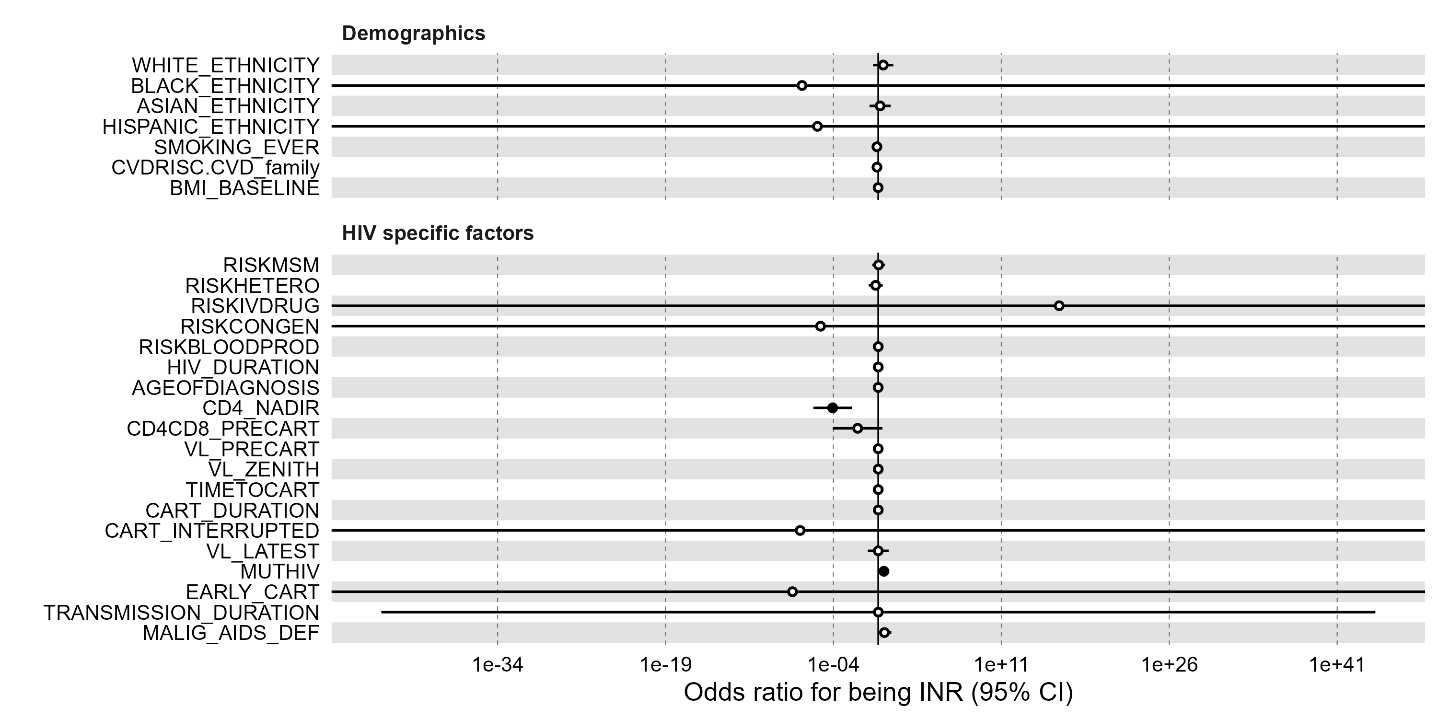


Supplementary figure 2E: Clinical associations of immunological non-responders compared to immunological responders as predictor for comorbidities and medical drug use. Multivariate logistic regression analysis with sex and age as covariates in the discovery cohort.


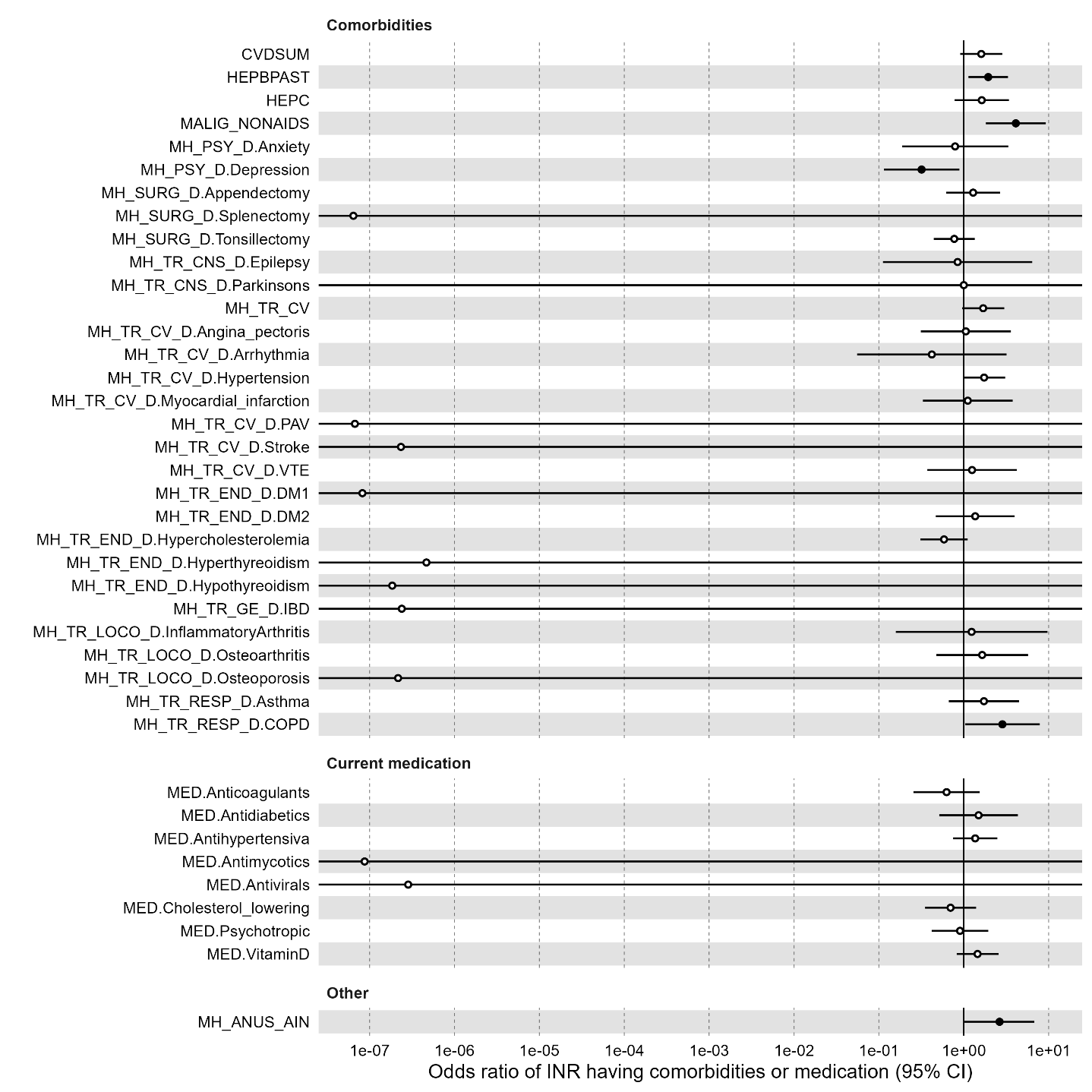


Supplementary figure 2F: Clinical associations of INR as predictor for comorbidities and medical drug use. Multivariate logistic regression analysis with sex and age as covariates in het validation cohort.


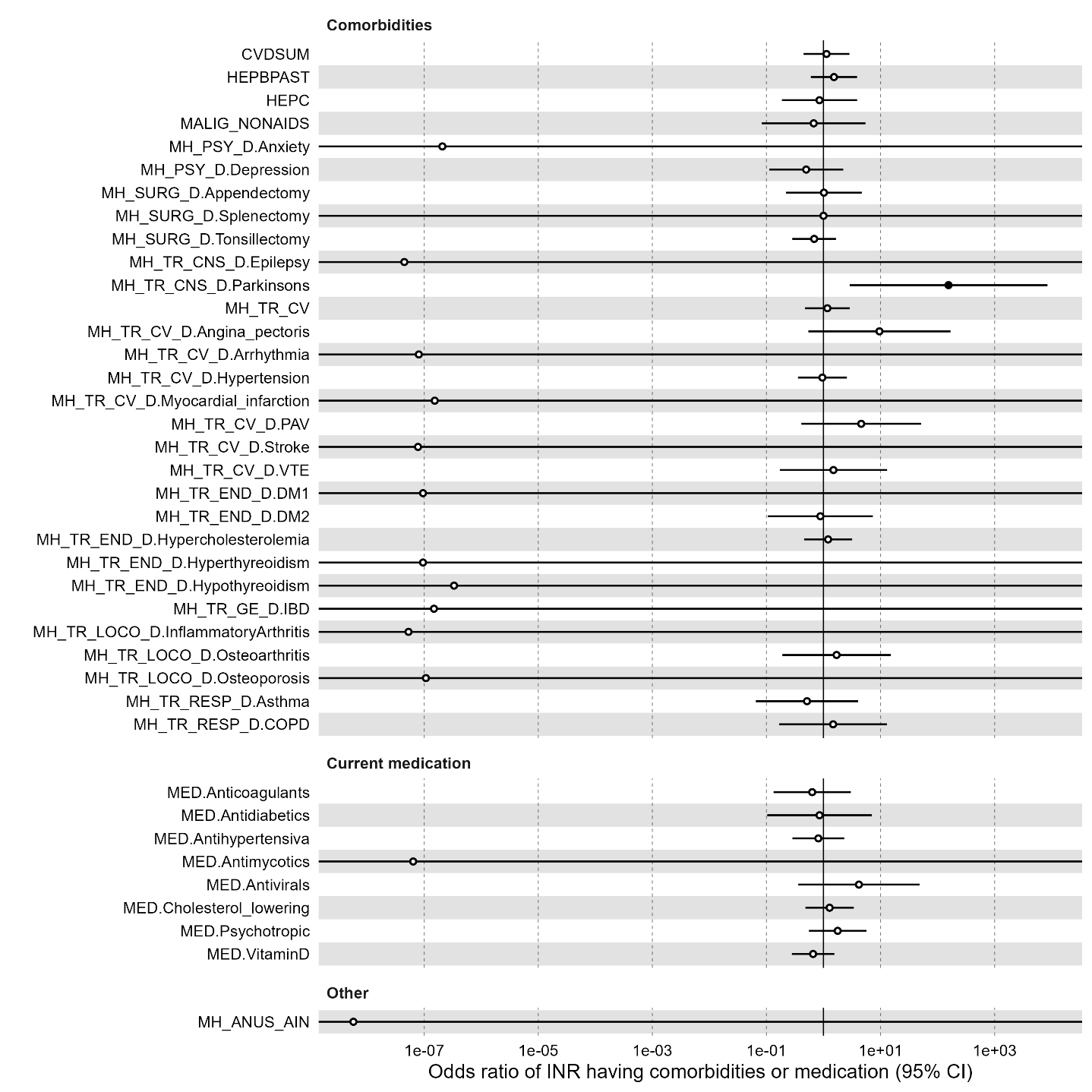


Supplementary figure 2G: Associations of combination antiretroviral regimens and immunological non-responders compared to immunological responders in the discovery cohort. Multivariate logistic regression analysis with sex and age as covariates.


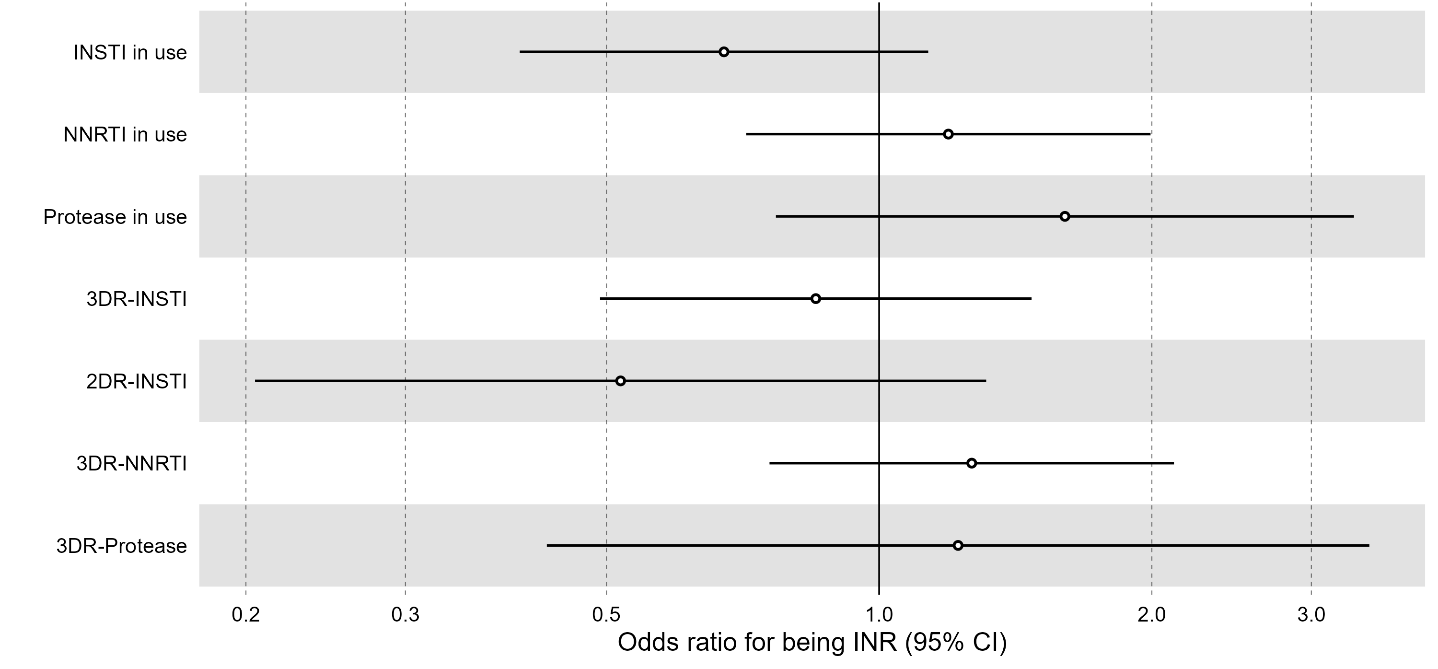


Abbreviations: INSTI: integrase strand transfer inhibitor; NNRTI: non-nucleoside reverse transcriptase inhibitor; protease: protease inhibitor; 3DR-INSTI: three antiretroviral drug regimens with an integrase strand transfer inhibitor in use; 2DR-INSTI: two antiretroviral drug regimens with an integrase strand transfer inhibitor in use; 3DR-NNRTI: three antiretroviral drug regimens with an non-nucleoside reverse transcriptase inhibitor in use; 3DR-Protease: three antiretroviral drug regimens with an protease inhibitor in use;

Supplementary figure 2H: Associations of combination antiretroviral regimens and immunological non-responders compared to immunological responders in the validation cohort. Multivariate logistic regression analysis with sex and age as covariates.


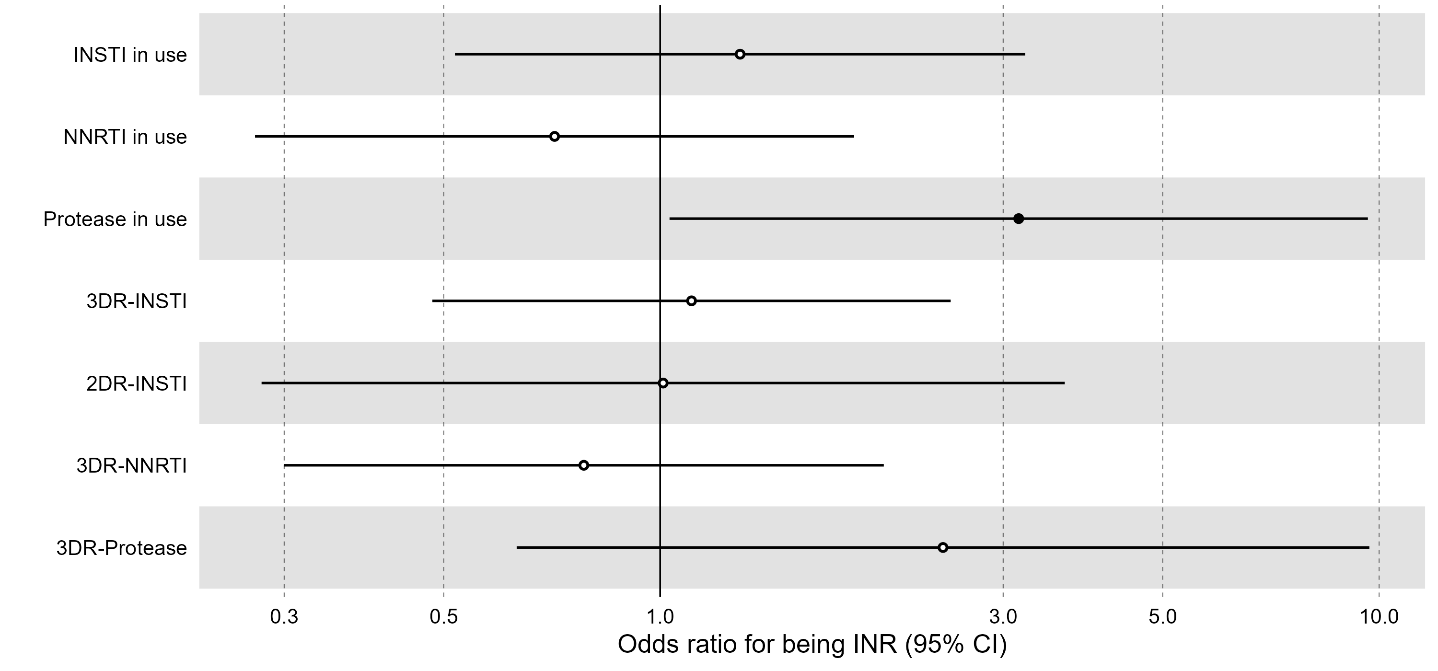


Abbreviations: INSTI: integrase strand transfer inhibitor; NNRTI: non-nucleoside reverse transcriptase inhibitor; protease: protease inhibitor; 3DR-INSTI: three antiretroviral drug regimens with an integrase strand transfer inhibitor in use; 2DR-INSTI: two antiretroviral drug regimens with an integrase strand transfer inhibitor in use; 3DR-NNRTI: three antiretroviral drug regimens with an non-nucleoside reverse transcriptase inhibitor in use; 3DR-Protease: three antiretroviral drug regimens with an protease inhibitor in use;

**Supplementary figure 3:** Flowchart showing population definitions used in flow cytometry analysis.
**Supplementary figure 3A:** Definitions of all flow cytometry populations panel 1

**Supplementary figure 3B:** Definitions of all flow cytometry populations panel 2

**Supplementary figure 3C:** Definitions of all flow cytometry populations panel 3


**Supplementary figure 4:** Flow cytometry of all measured cell populations comparing INR to IR. Linear model using sex, age, seasonality and COVID vaccination as covariates. Estimates are taken from the discovery cohort.
Supplementary figure 4A: Absolute counts of all cell populations comparing INR to IR.
** Indicates significant difference in both the discovery and validation cohort. *^ indicates significance in the discovery cohort and equal directionality change in the validation cohort without significance. *# indicates significance in the discovery cohort but with contradictory directionality change in validation cohort.

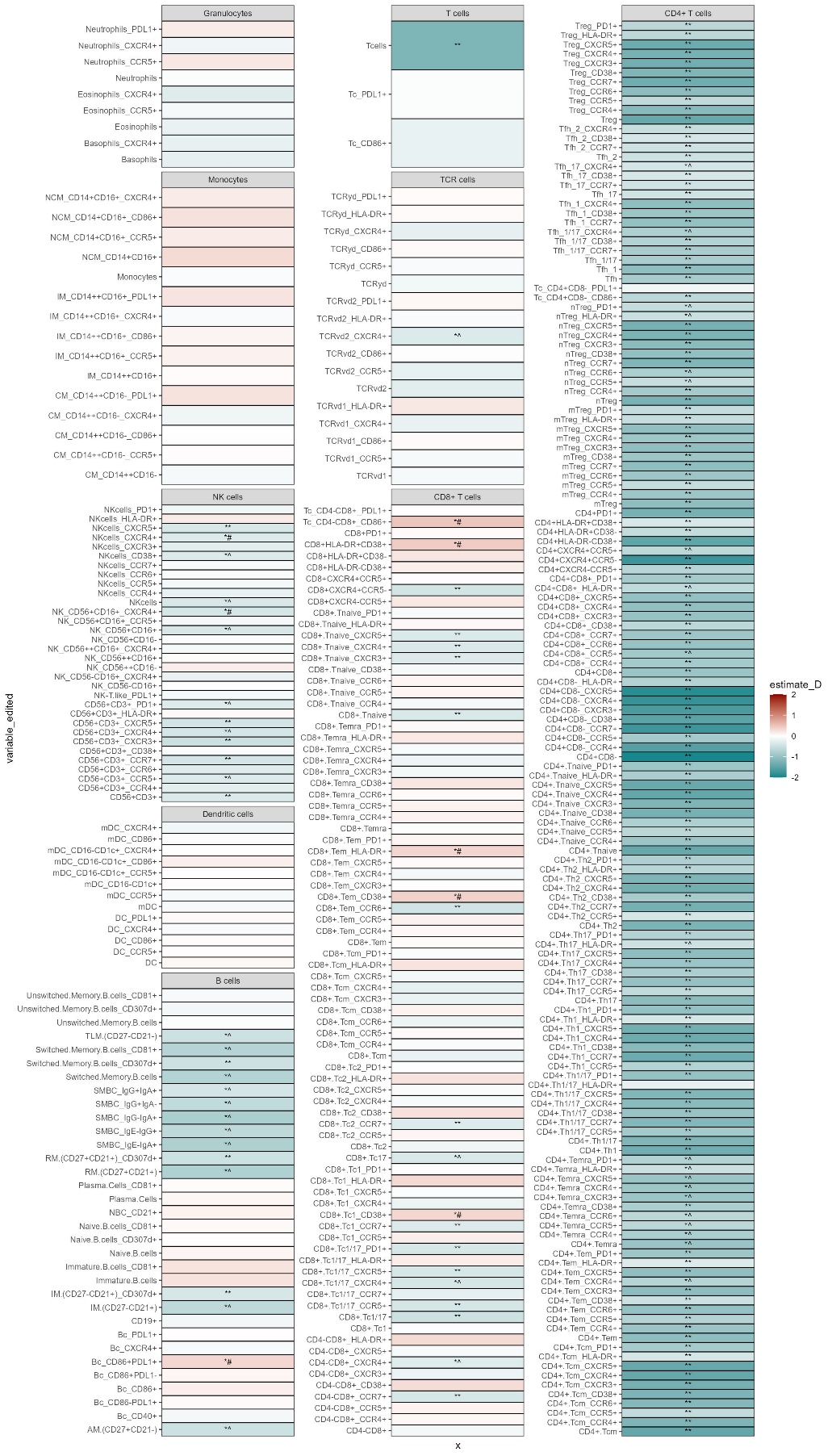


Supplementary figure 4B: Percentage counts of all cell populations comparing INR to IR.
** Indicates significant difference in both the discovery and validation cohort. *^ indicates significance in the discovery cohort and equal directionality change in the validation cohort without significance. *# indicates significance in the discovery cohort but with contradictory directionality change in validation cohort.

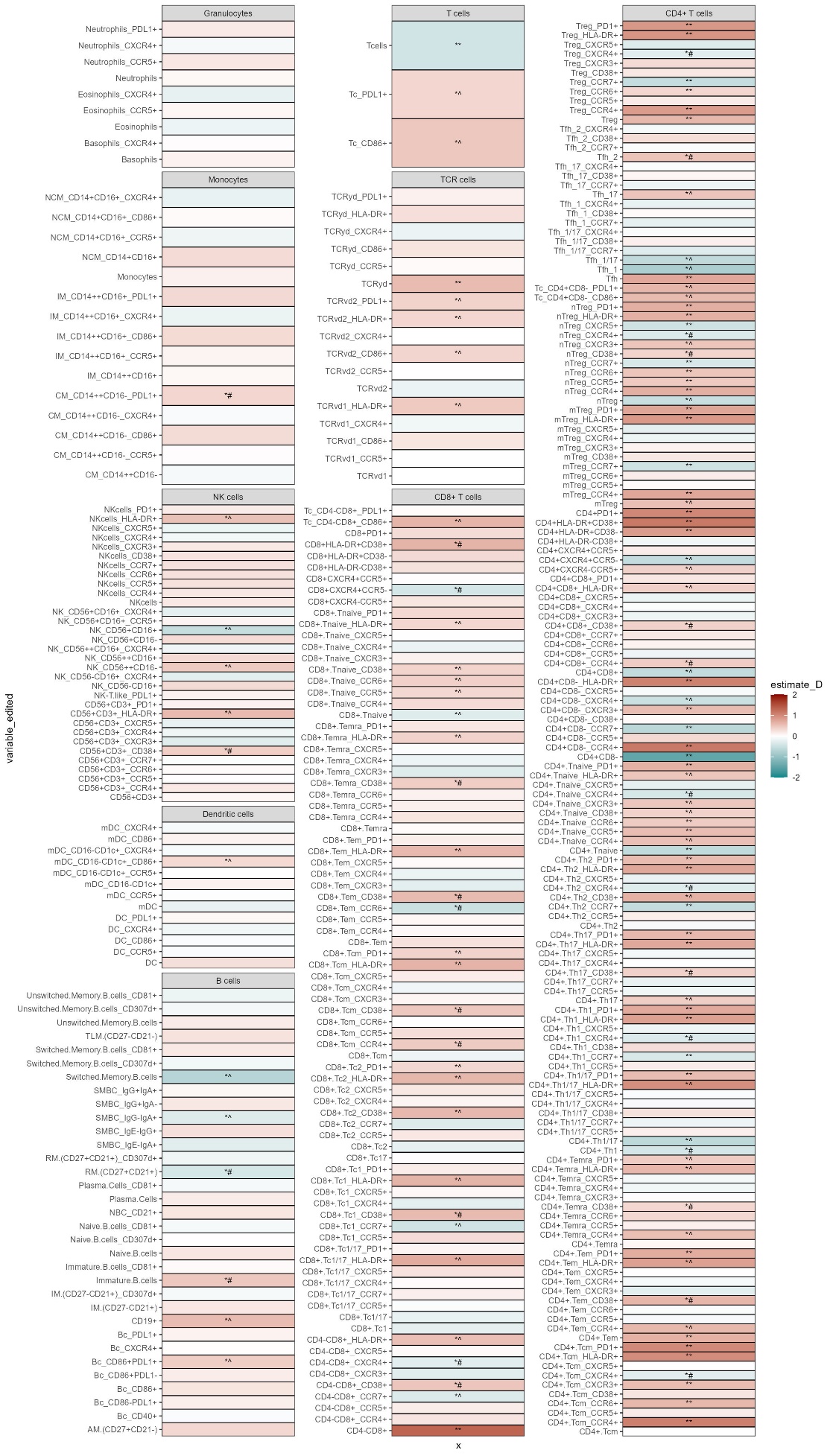


Supplementary figure 4C: Relative percentages of NK cell subpopulations in immunological responders compared to immunological responders in the discovery and validation cohort. Y-axis shows the main cell populations, X-axis displays the receptor that is added to the main population to depict the subpopulation. Estimates shown are from the discovery cohort.

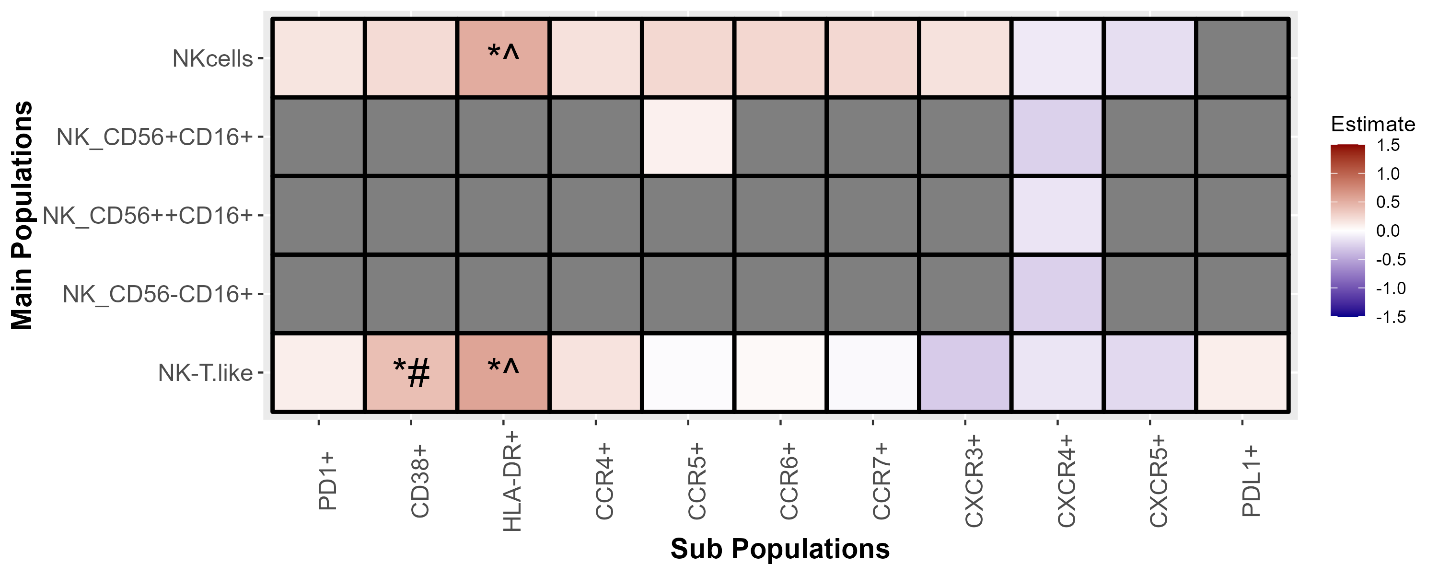


Supplementary figure 4D: Relative percentages of innate immune cell subpopulations in immunological non-responders compared to immunological responders in the discovery and validation cohort, with the exception of NK cells (sup figure 3C). Y-axis shows the main cell populations, X-axis displays the receptor that is added to the main population to depict the subpopulation. Estimates shown are from the discovery cohort.


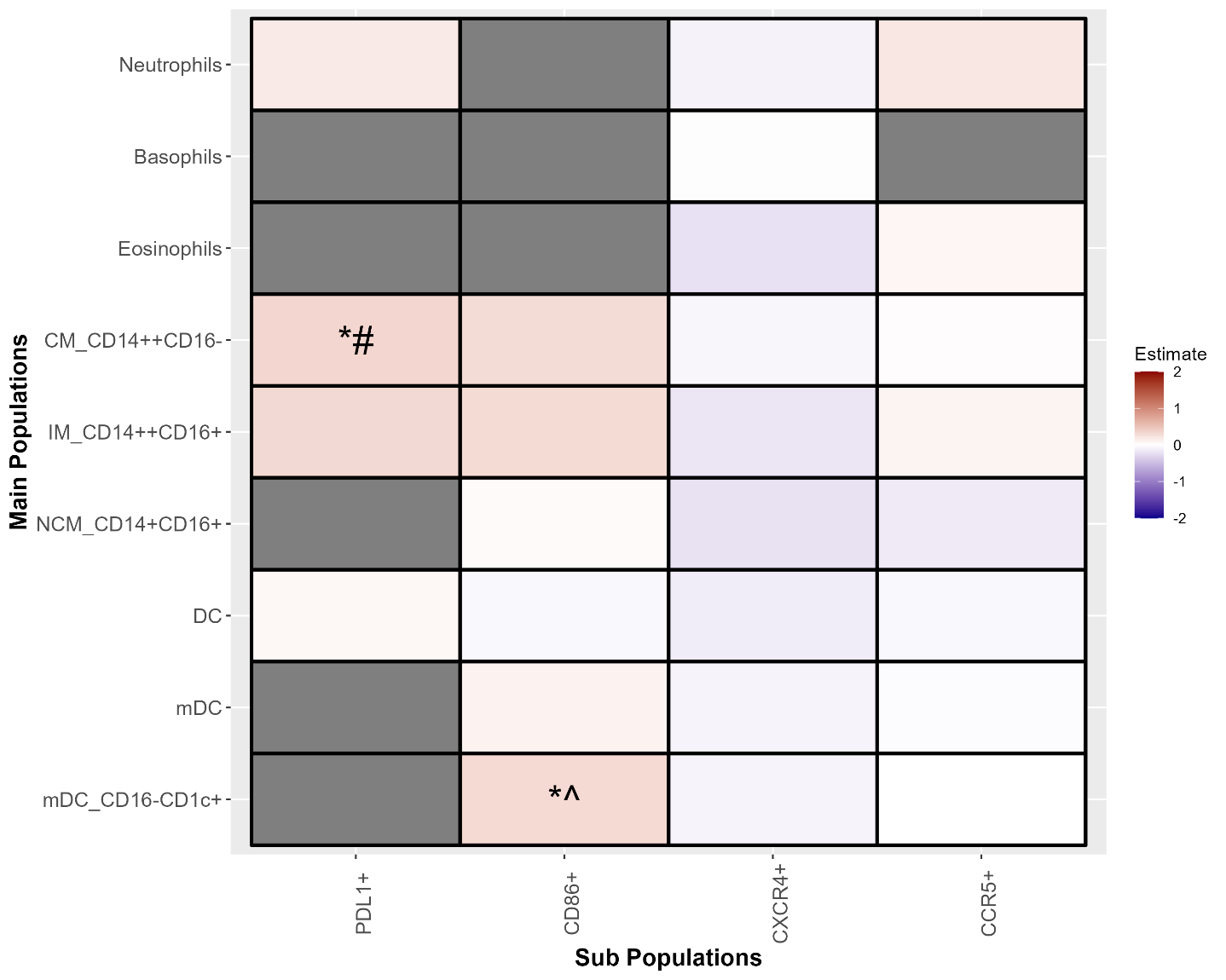


Supplementary figure 4E: Mean fluorescence intensity (MFI) of PD1, CD38 and HLA-DR in CD4+ T-cells in immunological non-responders compared to immunological responders in the discovery and validation cohorts. Y-axis shows the main CD4 T-cell populations, X-axis displays receptors of which the MFI is displayed in the heatmap. Estimates shown are from the discovery cohort.


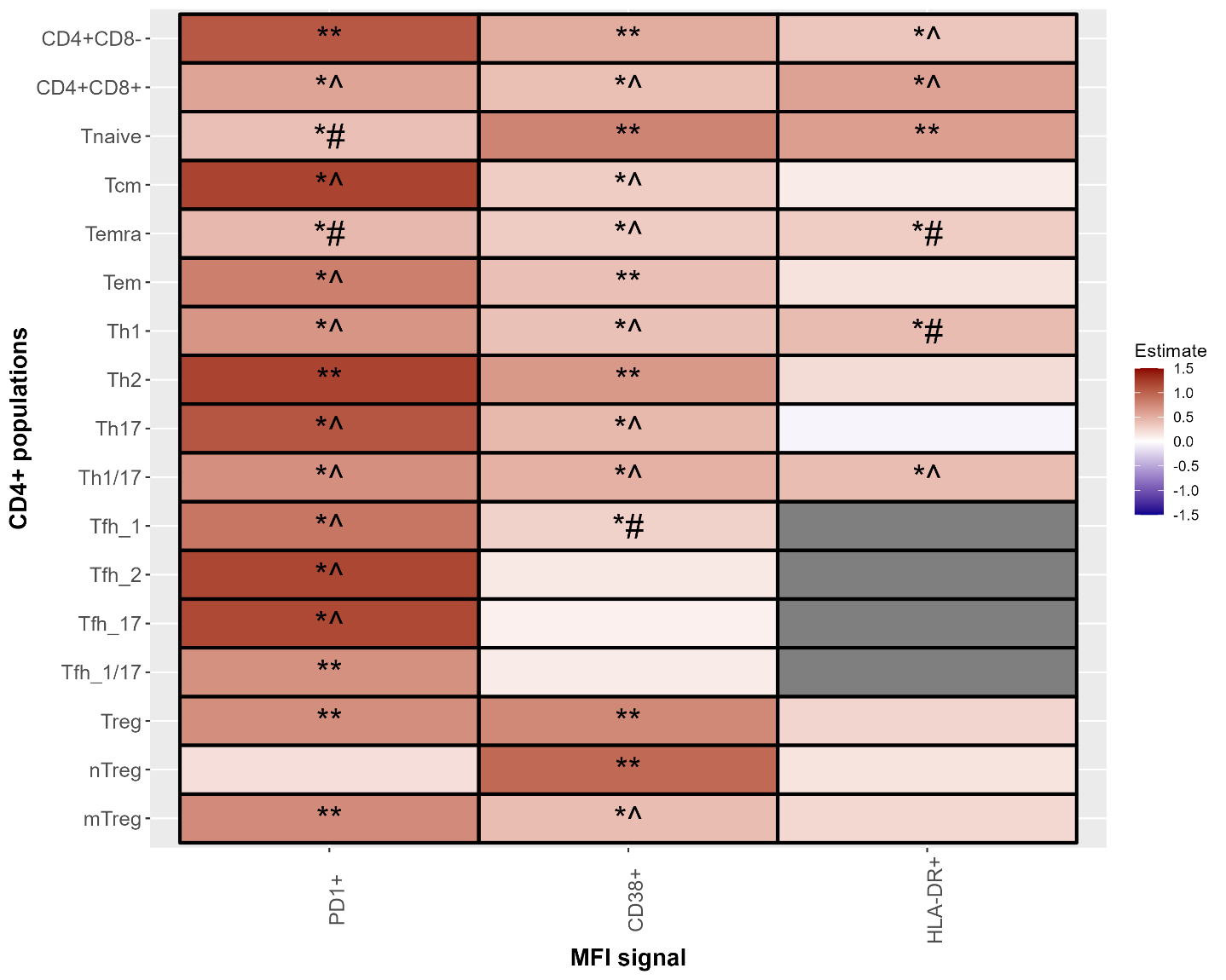


Abbreviations: CM: classical monocytes; DC: dendritic cells; IM: intermediate monocytes; mDC: myelodendritic cells; Mono: monocytes; NCM: non-classical monocytes; NK cells: natural killer cells; NK-T.like: Naturel Killer T-like cells

**Supplementary figure 5:** Correlation between proportions flow cytometry CD4+ subpopulations of activation (CD38+HLA-DR+) and exhaustion (PD1+) and peripheral blood mononuclear cell cytokine production after 7 days of stimulation. Linear model using age, sex, seasonality, past COVID-19 vaccination and lymphocyte/monocyte ratio as covariates.


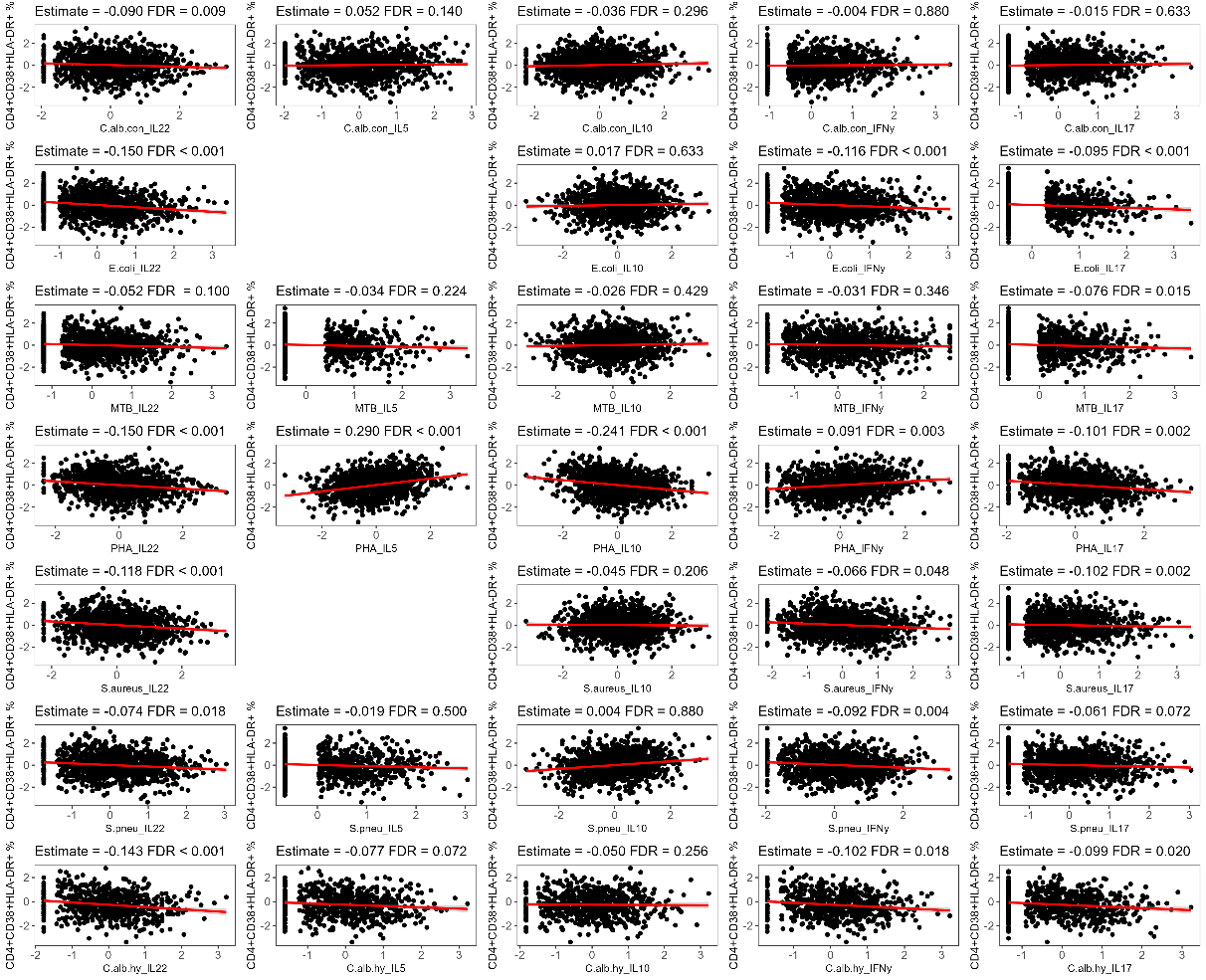
Supplementary figure 5A: Correlation between the proportion of CD4+CD38+HLA-DR+ subpopulations and cytokine production after 7 days of stimulation using the full discovery cohort.

Supplementary figure 5B: Correlation between the proportion of CD4+CD38+HLA-DR+
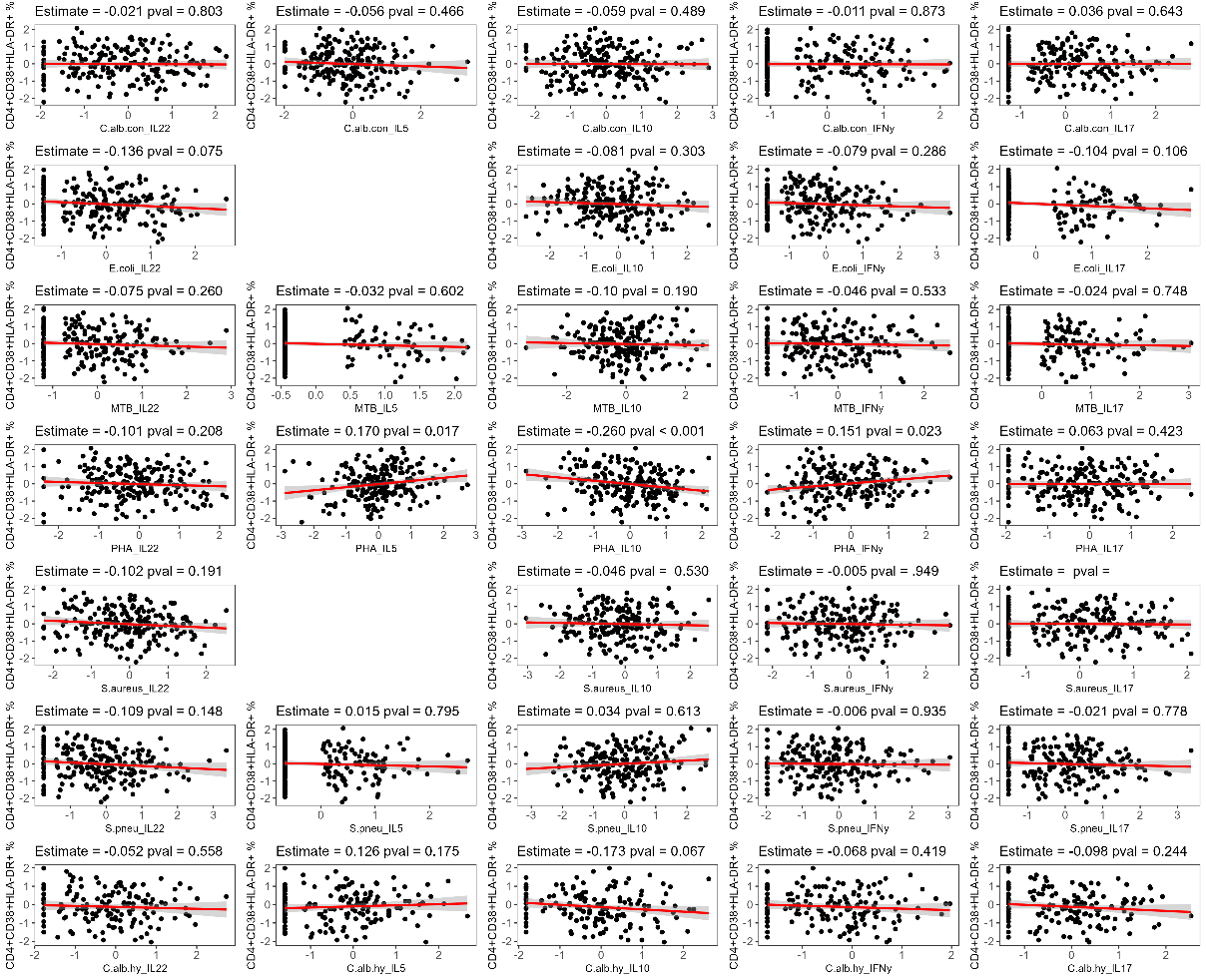
subpopulations and cytokine production after 7 days of stimulation using the full validation cohort.

Supplementary figure 5C: Correlation between the proportion of CD4+PD1+ subpopulations and
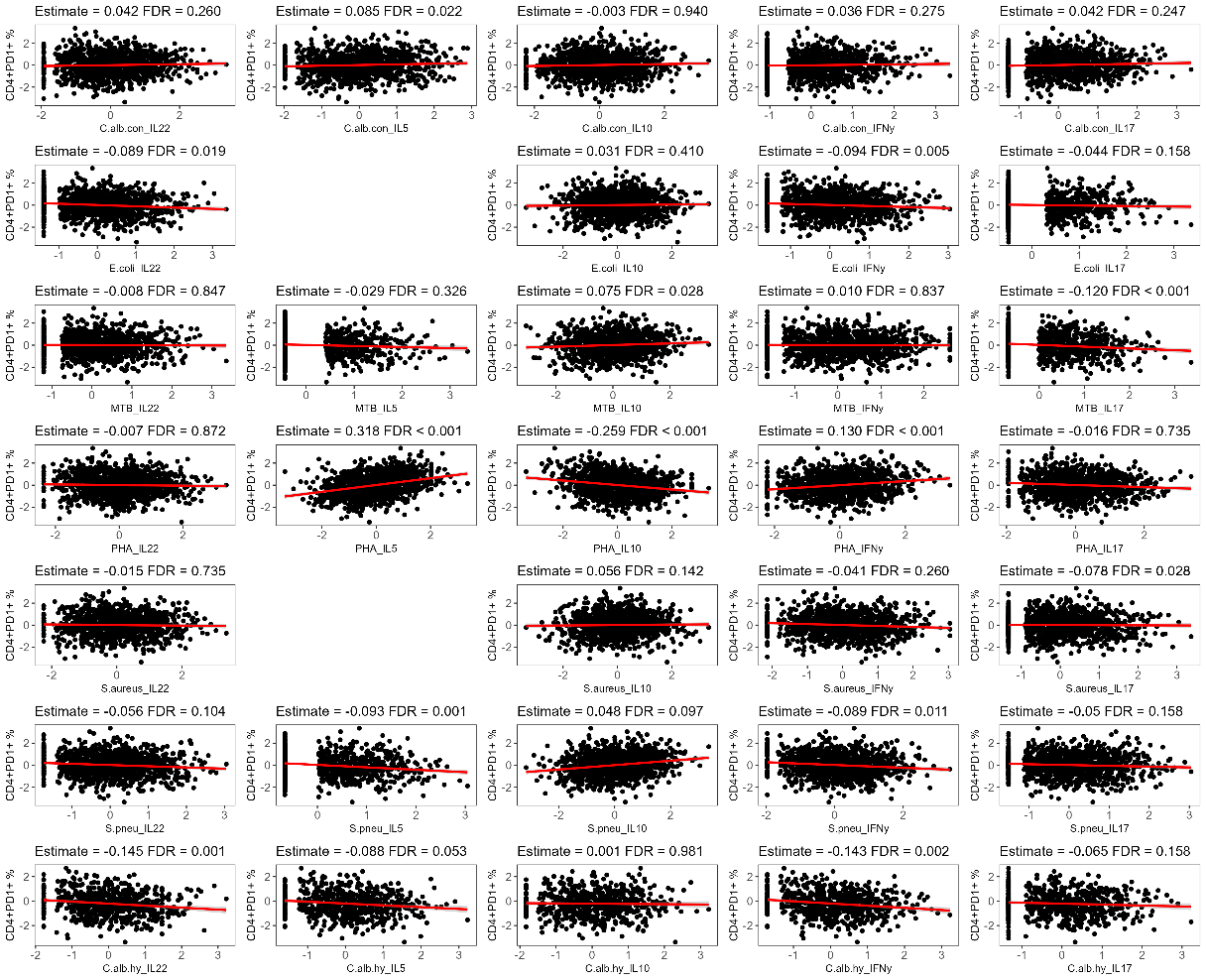
cytokine production after 7 days of stimulation using the full discovery cohort.

Supplementary figure 5D: Correlation between the proportion of CD4+HLA-DR+ subpopulations and
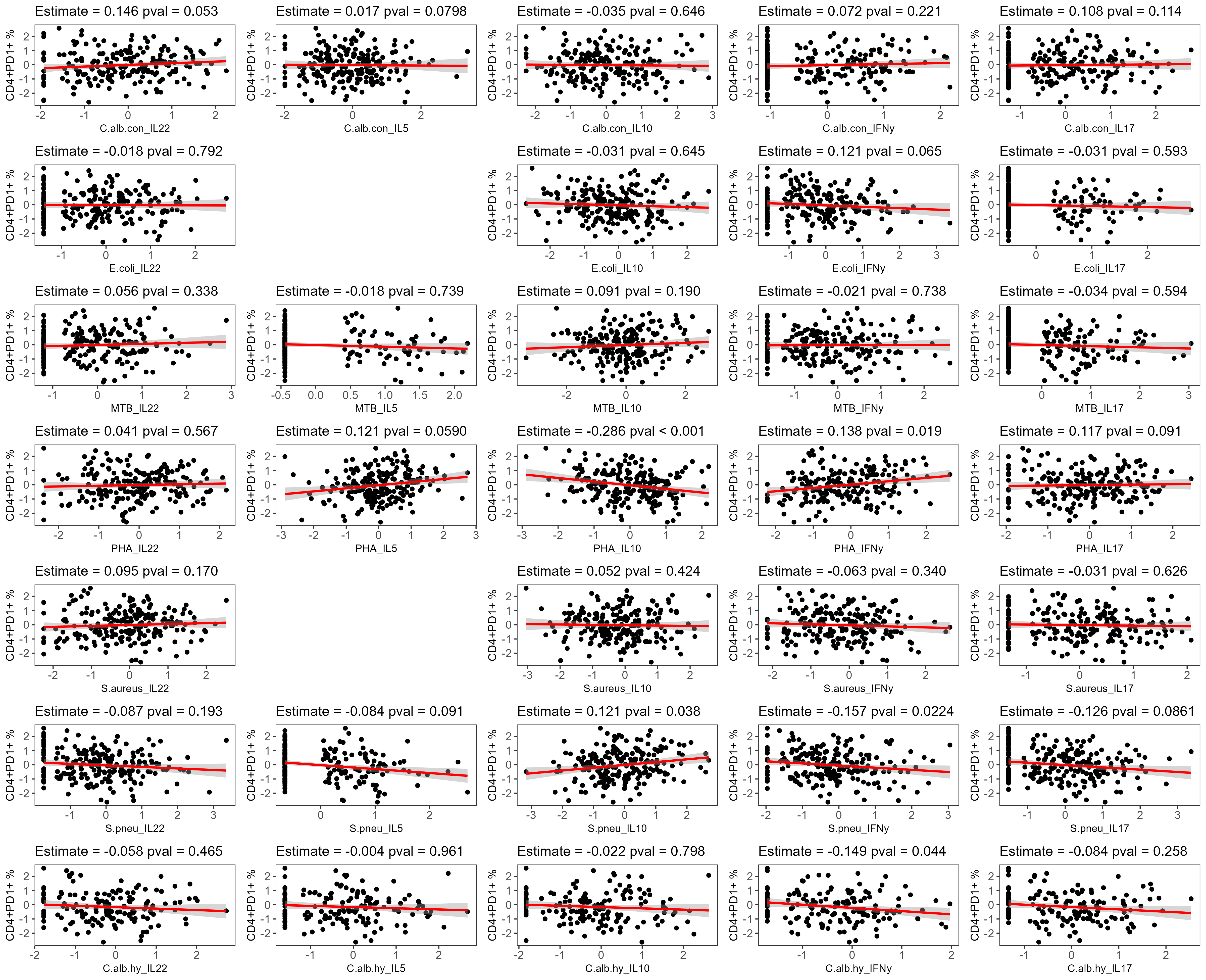
cytokine production after 7 days of stimulation using the full validation cohort.

**Supplementary table 1:** Baseline characteristics
Supplementary table 1A: Baseline characteristics discovery cohort

|  | Non-Responder (n=62) | Responder (n=1224) |
| --- | --- | --- |
| Age (Years) | 55.5 (47.0 - 63.0) | 52.0 (42.8 - 59.0) |
| Sex assigned at birth | | |
| Male | 52 (83.9%) | 1,036 (84.6%) |
| Female | 10 (16.1%) | 188 (15.4%) |
| Ethnicity | | |
| Non-white | 24 (38.7%) | 318 (26.0%) |
| White | 38 (61.3%) | 906 (74.0%) |
| Ethnicity | | |
| Non-black | 45 (72.6%) | 1,105 (90.3%) |
| Black | 17 (27.4%) | 119 (9.7%) |
| Viral Load Zenith (copies/ml) | | |
| Median (IQR) | 668.0 (380.0 - 902.8) | 634.0 (342.0 - 875.0) |
| Missing | 10 (16.1%) | 91 (7.4%) |
| CD4 Nadir (x10^9 cells/L) | | |
| Median (IQR) | 33.0 (12.0 - 52.0) | 100.0 (63.0 - 141.0) |
| Missing | 3 (4.8%) | 22 (1.8%) |
| Time since HIV diagnosis (Years) | 12.2 (6.7 - 18.9) | 13.1 (8.2 - 19.6) |
| cART Duration (Years) | | |
| Median (IQR) | 11.3 (6.2 - 16.6) | 10.5 (6.5 - 16.5) |
| Missing | 0 (0%) | 1 (0.1%) |
| Currently smoking | | |
| No | 40 (64.5%) | 774 (63.2%) |
| Yes | 16 (25.8%) | 376 (30.7%) |
| Missing | 6 (9.7%) | 74 (6.0%) |
| BMI | 24.8 (22.0 - 27.6) | 24.9 (22.5 - 27.7) |
| Cardiovascular disease | | |
| No | 56 (90.3%) | 1,113 (90.9%) |
| Yes | 6 (9.7%) | 111 (9.1%) |
| Myocardial infarction | | |
| No | 59 (95.2%) | 1,180 (96.4%) |
| Yes | 3 (4.8%) | 44 (3.6%) |
| Stroke | | |
| No | 62 (100.0%) | 1,187 (97.0%) |
| Yes | 0 (0.0%) | 37 (3.0%) |
| Peripheral arterial disease | | |
| No | 62 (100.0%) | 1,217 (99.4%) |
| Yes | 0 (0.0%) | 7 (0.6%) |
| Angina pectoris | | |
| No | 59 (95.2%) | 1,180 (96.4%) |
| Yes | 3 (4.8%) | 44 (3.6%) |
| Had Non-AIDS malignancy | | |
| No | 52 (83.9%) | 1,177 (96.2%) |
| Yes | 9 (14.5%) | 42 (3.4%) |
| Missing | 1 (1.6%) | 5 (0.4%) |
| Past HBV | | |
| No | 29 (46.8%) | 815 (66.6%) |
| Yes | 33 (53.2%) | 409 (33.4%) |

Supplementary table 1B: Baseline characteristics validation cohort

|  | Non-Responder (n=26) | Responder (n=243) |
| --- | --- | --- |
| Age (Years) | 54.0 (48.5 - 59.8) | 53.0 (47.0 - 61.0) |
| Sex at birth | | |
| Male | 25 (96.2%) | 198 (81.5%) |
| Female | 1 (3.8%) | 45 (18.5%) |
| Ethnicity | | |
| Non-white | 1 (3.8%) | 34 (14.0%) |
| White | 25 (96.2%) | 209 (86.0%) |
| Ethnicity | | |
| Non-black | 26 (100.0%) | 223 (91.8%) |
| Black | 0 (0.0%) | 20 (8.2%) |
| Viral Load Zenith (copies/ml) | | |
| Median (IQR) | 1015.0 (702.5 - 1088.8) | 729.0 (377.0 - 940.0) |
| Missing | 4 (15.4%) | 24 (9.9%) |
| CD4 Nadir (x10^9 cells/L) | | |
| Median (IQR) | 40.0 (31.0 - 55.0) | 107.0 (73.0 - 153.5) |
| Missing | 1 (3.8%) | 8 (3.3%) |
| Time since HIV diagnosis (Years) | 9.8 (4.8 - 18.0) | 10.9 (5.8 - 16.7) |
| cART Duration (Years) | 7.9 (3.9 - 16.6) | 8.7 (5.2 - 14.1) |
| Currently smoking | | |
| No | 17 (65.4%) | 148 (60.9%) |
| Yes | 5 (19.2%) | 75 (30.9%) |
| Missing | 4 (15.4%) | 20 (8.2%) |
| BMI | 25.2 (23.1 - 26.5) | 25.7 (23.0 - 27.9) |
| Myocardial infarction | | |
| No | 26 (100.0%) | 228 (93.8%) |
| Yes | 0 (0.0%) | 15 (6.2%) |
| Stroke | | |
| No | 26 (100.0%) | 233 (95.9%) |
| Yes | 0 (0.0%) | 10 (4.1%) |
| Peripheral artery disease | | |
| No | 25 (96.2%) | 240 (98.8%) |
| Yes | 1 (3.8%) | 3 (1.2%) |
| Angina pectoris | | |
| No | 25 (96.2%) | 242 (99.6%) |
| Yes | 1 (3.8%) | 1 (0.4%) |
| Had Non-AIDS malignancy | | |
| No | 25 (96.2%) | 228 (93.8%) |
| Yes | 1 (3.8%) | 15 (6.2%) |
| Past HBV | | |
| No | 16 (61.5%) | 176 (72.4%) |
| Yes | 10 (38.5%) | 67 (27.6%) |

Abbreviations: BMI: body mass index; cART: combination antiretroviral therapy; HBV: hepatitis B virus; IQR: interquartile range

**Supplementary table 2: Clinical associations of immunological non-responders**
Supplementary table 2A: Clinical associations of sex and age in relation to immunological non-responders (INR) compared to immunological responders (IR). Univariate logistic regression analysis in the discovery cohort.

| \| name \| outcome \| beta \| se \| z value \| Pr(>\|z\|) \| 2.5 % \| 97.5 % \| OR \| \| --- \| --- \| --- \| --- \| --- \| --- \| --- \| --- \| --- \| \| Sex \| INR \| 0,058 \| 0,354 \| 0,164 \| 0,870 \| 0,529 \| 2,122 \| 1,060 \| \| Age \| INR \| 0,024 \| 0,011 \| 2,145 \| 0,032 \| 1,002 \| 1,048 \| 1,025 \| |  |  |  |  |  |  |  |  |  |
| --- | --- | --- | --- | --- | --- | --- | --- | --- | --- | --- | --- | --- | --- | --- | --- | --- | --- | --- | --- | --- | --- | --- | --- | --- | --- | --- | --- | --- | --- | --- | --- | --- | --- | --- | --- | --- |

Supplementary table1B: Clinical associations of sex and age in relation to immunological non-responders (INR) compared to immunological responders (IR). Univariate logistic regression analysis in the validation cohort.

| \| name \| outcome \| beta \| se \| z value \| Pr(>\|z\|) \| 2.5 % \| 97.5 % \| OR \| \| --- \| --- \| --- \| --- \| --- \| --- \| --- \| --- \| --- \| \| Sex \| INR \| -1,737 \| 1,033 \| -1,682 \| 0,093 \| 0,023 \| 1,333 \| 0,176 \| \| Age \| INR \| 0,004 \| 0,020 \| 0,195 \| 0,845 \| 0,966 \| 1,043 \| 1,004 \| |  |  |  |  |  |  |  |  |  |
| --- | --- | --- | --- | --- | --- | --- | --- | --- | --- | --- | --- | --- | --- | --- | --- | --- | --- | --- | --- | --- | --- | --- | --- | --- | --- | --- | --- | --- | --- | --- | --- | --- | --- | --- | --- | --- |

Supplementary table 2C: Clinical factors predictive of INR phenotype compared to IR phenotype in the discovery cohort. Multivariate logistic regression analysis with sex and age as covariates.

| name | outcome | beta | se | z value | Pr(>\|z\|) | 2.5 % | 97.5 % | OR |
| --- | --- | --- | --- | --- | --- | --- | --- | --- |
| White ethnicity | INR | -0,863 | 0,292 | -2,959 | 0,003 | 0,238 | 0,747 | 0,422 |
| Black ethnicity | INR | 1,625 | 0,336 | 4,836 | 0,000 | 2,628 | 9,808 | 5,077 |
| Asian ethnicity | INR | 0,446 | 0,538 | 0,829 | 0,407 | 0,544 | 4,482 | 1,562 |
| Hispanic ethnicity | INR | -0,484 | 1,027 | -0,471 | 0,638 | 0,082 | 4,612 | 0,617 |
| Has ever smoked | INR | 0,246 | 0,298 | 0,825 | 0,409 | 0,713 | 2,294 | 1,279 |
| CVD in family at young age | INR | -0,298 | 0,341 | -0,875 | 0,382 | 0,381 | 1,447 | 0,742 |
| BMI | INR | -0,029 | 0,033 | -0,872 | 0,383 | 0,911 | 1,036 | 0,972 |
| MSM HIV infection | INR | -1,369 | 0,345 | -3,968 | 0,000 | 0,129 | 0,500 | 0,254 |
| Heterosexual HIV infection | INR | 0,863 | 0,396 | 2,180 | 0,029 | 1,091 | 5,152 | 2,371 |
| IV drug HIV infection | INR | 2,679 | 0,611 | 4,383 | 0,000 | 4,397 | 48,285 | 14,571 |
| Congenital HIV infection | INR | -11,863 | 593,869 | -0,020 | 0,984 | 0,000 | #GETAL! | 0,000 |
| Blood product HIV infection | INR | -12,567 | 725,231 | -0,017 | 0,986 | 0,000 | #GETAL! | 0,000 |
| Duration of HIV infection | INR | -0,039 | 0,018 | -2,108 | 0,035 | 0,928 | 0,997 | 0,962 |
| Age at time of HIV diagnosis | INR | 0,039 | 0,018 | 2,108 | 0,035 | 1,003 | 1,078 | 1,040 |
| CD4 Nadir | INR | -12,088 | 1,541 | -7,844 | 0,000 | 0,000 | 0,000 | 0,000 |
| Last CD4/CD8 ratio before start cART | INR | -8,579 | 1,388 | -6,179 | 0,000 | 0,000 | 0,003 | 0,000 |
| Last viral load before start cART | INR | 0,000 | 0,000 | -0,944 | 0,345 | 1,000 | 1,000 | 1,000 |
| Viral load zenith | INR | 0,000 | 0,000 | -0,557 | 0,577 | 1,000 | 1,000 | 1,000 |
| Time between HIV diagnosis and start cART | INR | -0,001 | 0,000 | -2,572 | 0,010 | 0,999 | 1,000 | 0,999 |
| Time on cART | INR | -0,021 | 0,022 | -0,949 | 0,342 | 0,938 | 1,022 | 0,979 |
| Was cART ever (temporarily) stopped | INR | 0,515 | 0,396 | 1,301 | 0,193 | 0,770 | 3,637 | 1,674 |
| Latest viral load | INR | -0,343 | 1,028 | -0,334 | 0,738 | 0,095 | 5,317 | 0,710 |
| Latest CD4/CD8 ratio | INR | -5,614 | 0,673 | -8,348 | 0,000 | 0,001 | 0,014 | 0,004 |
| HIV strain has at least one drug resistance mutation | INR | 0,392 | 0,511 | 0,767 | 0,443 | 0,543 | 4,034 | 1,481 |
| Start cART<6 months after infection | INR | -16,325 | 1355,968 | -0,012 | 0,990 | 0,000 | #GETAL! | 0,000 |
| Time between HIV infection and start cART | INR | -0,001 | 0,001 | -0,893 | 0,372 | 0,996 | 1,002 | 0,999 |
| AIDS defining malignancy | INR | 0,724 | 0,427 | 1,696 | 0,090 | 0,893 | 4,764 | 2,063 |
| AIDS defining disease | INR | 1,217 | 27,06 | 4,499 | 0,000 | 1,988 | 5,743 | 3,379 |

Supplementary table 2C: Clinical factors predictive of INR phenotype compared to IR phenotype in the validation cohort. Multivariate logistic regression analysis with sex and age as covariates.

| name | outcome | beta | se | z value | Pr(>\|z\|) | 2.5 % | 97.5 % | OR |
| --- | --- | --- | --- | --- | --- | --- | --- | --- |
| White ethnicity | INR | 1,040 | 1,056 | 0,985 | 0,325 | 0,357 | 22,436 | 2,830 |
| Black ethnicity | INR | -15,670 | 1420,405 | -0,011 | 0,991 | 0,000 | INF | 0,000 |
| Asian ethnicity | INR | 0,393 | 1,105 | 0,355 | 0,722 | 0,170 | 12,912 | 1,481 |
| Hispanic ethnicity | INR | -12,499 | 882,743 | -0,014 | 0,989 | 0,000 | INF | 0,000 |
| Has ever smoked | INR | -0,263 | 0,469 | -0,561 | 0,575 | 0,307 | 1,927 | 0,769 |
| CVD in family at young age | INR | -0,252 | 0,524 | -0,481 | 0,630 | 0,278 | 2,170 | 0,777 |
| BMI | INR | -0,020 | 0,053 | -0,384 | 0,701 | 0,884 | 1,087 | 0,980 |
| MSM HIV infection | INR | 0,081 | 0,653 | 0,125 | 0,901 | 0,302 | 3,904 | 1,085 |
| Heterosexual HIV infection | INR | -0,516 | 0,732 | -0,705 | 0,481 | 0,142 | 2,506 | 0,597 |
| IV drug HIV infection | INR | 37,223 | 6596,263 | 0,006 | 0,995 | 0,000 | INF | INF |
| Congenital HIV infection | INR | -11,862 | 1029,121 | -0,012 | 0,991 | 0,000 | INF | 0,000 |
| Blood product HIV infection | INR | -0,001 | 0,021 | -0,030 | 0,976 |  |  | 0,999 |
| Duration of HIV infection | INR | 0,008 | 0,029 | 0,292 | 0,770 | 0,953 | 1,068 | 1,009 |
| Age at time of HIV diagnosis | INR | -0,008 | 0,029 | -0,292 | 0,770 | 0,937 | 1,050 | 0,992 |
| CD4 Nadir | INR | -9,373 | 2,013 | -4,657 | 0,000 | 0,000 | 0,004 | 0,000 |
| Last viral load before start cART | INR | -4,226 | 2,602 | -1,624 | 0,104 | 0,000 | 2,395 | 0,015 |
| Viral load zenith | INR | 0,000 | 0,000 | 0,194 | 0,846 | 1,000 | 1,000 | 1,000 |
| Time between HIV diagnosis and start cART | INR | 0,000 | 0,000 | -0,114 | 0,909 | 1,000 | 1,000 | 1,000 |
| Time on cART | INR | 0,000 | 0,000 | -0,809 | 0,419 | 0,999 | 1,000 | 1,000 |
| Was cART ever (temporarily) stopped | INR | 0,010 | 0,033 | 0,307 | 0,759 | 0,947 | 1,078 | 1,010 |
| Latest viral load | INR | -16,055 | 1347,033 | -0,012 | 0,990 | 0,000 | INF | 0,000 |
| Latest CD4/CD8 ratio | INR | 0,007 | 1,093 | 0,007 | 0,995 | 0,118 | 8,582 | 1,007 |
| HIV strain has at least one drug resistance mutation | INR | 1,169 | 0,562 | 2,079 | 0,038 | 1,069 | 9,695 | 3,220 |
| Start cART<6 months after infection | INR | -17,631 | 3605,233 | -0,005 | 0,996 | 0,000 | INF | 0,000 |
| Time between HIV infection and start cART | INR | 0,025 | 52,160 | 0,000 | 1,000 | 0,000 | INF | 1,025 |
| AIDS defining malignancy | INR | 1,284 | 0,720 | 1,783 | 0,075 | 0,881 | 14,803 | 3,611 |

Supplementary table 2E: Clinical associations of INR as predictor for comorbidities and medical drug use in the discovery cohort. Multivariate logistic regression analysis with sex and age as covariates.

| outcome | name | beta | se | z value | Pr(>\|z\|) | 2.5 % | 97.5 % | OR |
| --- | --- | --- | --- | --- | --- | --- | --- | --- |
| Cardiovascular disease (grouped) | INR | 0,475 | 0,292 | 1,628 | 0,104 | 0,908 | 2,846 | 1,607 |
| Previous hepatitis B infection | INR | 0,663 | 0,274 | 2,418 | 0,016 | 1,134 | 3,322 | 1,941 |
| Previous hepatitis C infection | INR | 0,488 | 0,376 | 1,299 | 0,194 | 0,780 | 3,400 | 1,629 |
| Non-AIDS malignancy | INR | 1,412 | 0,415 | 3,400 | 0,001 | 1,819 | 9,261 | 4,104 |
| Anticoagulants in use | INR | -0,467 | 0,458 | -1,018 | 0,309 | 0,255 | 1,540 | 0,627 |
| Antidiabetics in use | INR | 0,402 | 0,544 | 0,738 | 0,460 | 0,515 | 4,341 | 1,494 |
| Anti hypertensive medication in use | INR | 0,310 | 0,307 | 1,011 | 0,312 | 0,747 | 2,490 | 1,364 |
| Antimycotics in use | INR | -16,3 | 3600,5 | -0,005 | 0,996 | 0,000 | INF | 0,000 |
| Antiviral meds in use (other than cART) | INR | -15,1 | 827,9 | -0,018 | 0,985 | 0,000 | INF | 0,000 |
| Cholesterol lowering medication in use | INR | -0,359 | 0,353 | -1,015 | 0,310 | 0,349 | 1,397 | 0,699 |
| Psychotropic medication in use | INR | -0,102 | 0,392 | -0,260 | 0,795 | 0,419 | 1,946 | 0,903 |
| Vitamin D medication in use | INR | 0,374 | 0,289 | 1,292 | 0,196 | 0,824 | 2,562 | 1,453 |
| Anxiety disorder | INR | -0,235 | 0,735 | -0,320 | 0,749 | 0,187 | 3,337 | 0,790 |
| Depression | INR | -1,144 | 0,523 | -2,189 | 0,029 | 0,114 | 0,887 | 0,319 |
| Appendectomy | INR | 0,254 | 0,375 | 0,678 | 0,498 | 0,619 | 2,686 | 1,289 |
| Splenectomy | INR | -16,6 | 9347,0 | -0,002 | 0,999 | 0,000 | INF | 0,000 |
| Tonsillectomy | INR | -0,259 | 0,283 | -0,912 | 0,362 | 0,443 | 1,346 | 0,772 |
| Epilepsy | INR | -0,167 | 1,033 | -0,162 | 0,871 | 0,112 | 6,409 | 0,846 |
| Parkinsons disease | INR | 0,000 | 46443,4 | 0,000 | 1,000 | 0,000 | INF | 1,000 |
| Angina pectoris | INR | 0,056 | 0,624 | 0,090 | 0,929 | 0,311 | 3,590 | 1,057 |
| Arrythmia | INR | -0,866 | 1,034 | -0,838 | 0,402 | 0,055 | 3,193 | 0,420 |
| Hypertension | INR | 0,551 | 0,292 | 1,889 | 0,059 | 0,980 | 3,076 | 1,736 |
| Myocardial infarction | INR | 0,109 | 0,621 | 0,175 | 0,861 | 0,330 | 3,767 | 1,115 |
| Peripheral arterial disease | INR | -16,5 | 3404,2 | -0,005 | 0,996 | 0,000 | INF | 0,000 |
| Stroke | INR | -15,3 | 782,6 | -0,020 | 0,984 | 0,000 | INF | 0,000 |
| Venous thromboembolism | INR | 0,224 | 0,620 | 0,362 | 0,717 | 0,371 | 4,217 | 1,251 |
| Diabetes type 1 | INR | -16,313 | 5798,3 | -0,003 | 0,998 | 0,000 | INF | 0,000 |
| Diabetes type 2 | INR | 0,310 | 0,545 | 0,569 | 0,569 | 0,469 | 3,965 | 1,363 |
| Hypercholesterolemia | INR | -0,535 | 0,327 | -1,639 | 0,101 | 0,309 | 1,111 | 0,585 |
| Hyperthyroidism | INR | -14,6 | 1313,4 | -0,011 | 0,991 | 0,000 | INF | 0,000 |
| Hypothyreoidism | INR | -15,5 | 1309,0 | -0,012 | 0,991 | 0,000 | INF | 0,000 |
| Inflammatory bowel disease | INR | -15,2 | 1358,7 | -0,011 | 0,991 | 0,000 | INF | 0,000 |
| Inflammatory arthritis | INR | 0,212 | 1,050 | 0,202 | 0,840 | 0,158 | 9,682 | 1,237 |
| Osteoarthritis | INR | 0,498 | 0,636 | 0,784 | 0,433 | 0,473 | 5,722 | 1,646 |
| Osteoporosis | INR | -15,3 | 788,8 | -0,019 | 0,984 | 0,000 | INF | 0,000 |
| Asthma | INR | 0,547 | 0,487 | 1,124 | 0,261 | 0,666 | 4,485 | 1,728 |
| COPD | INR | 1,048 | 0,517 | 2,026 | 0,043 | 1,035 | 7,854 | 2,851 |
| High grade squamous intraepithelial anal lesions | INR | 0,970 | 0,483 | 2,007 | 0,045 | 1,023 | 6,805 | 2,638 |

Supplementary table 2F: Clinical associations of INR as predictor for comorbidities and medical drug use in the validation cohort. Multivariate logistic regression analysis with sex and age as covariates.

| outcome | name | beta | se | z value | Pr(>\|z\|) | 2.5 % | 97.5 % | OR |
| --- | --- | --- | --- | --- | --- | --- | --- | --- |
| Cardiovascular disease (grouped) | INR | 0,121 | 0,472 | 0,257 | 0,797 | 0,448 | 2,847 | 1,129 |
| Previous hepatitis B infection | INR | 0,422 | 0,476 | 0,888 | 0,375 | 0,600 | 3,877 | 1,526 |
| Previous hepatitis C infection | INR | -0,162 | 0,775 | -0,209 | 0,835 | 0,186 | 3,887 | 0,850 |
| Non-AIDS malignancy | INR | -0,396 | 1,067 | -0,371 | 0,711 | 0,083 | 5,448 | 0,673 |
| Anticoagulants in use | INR | -0,456 | 0,796 | -0,573 | 0,566 | 0,133 | 3,014 | 0,634 |
| Antidiabetics in use | INR | -0,159 | 1,077 | -0,148 | 0,882 | 0,103 | 7,042 | 0,853 |
| Anti hypertensive medication in use | INR | -0,208 | 0,534 | -0,389 | 0,697 | 0,285 | 2,315 | 0,813 |
| Antimycotics in use | INR | -16,56 | 5459 | -0,003 | 0,998 | 0,000 | INF | 0,000 |
| Antiviral meds in use (other than cART) | INR | 1,430 | 1,248 | 1,146 | 0,252 | 0,362 | 48 | 4,178 |
| Cholesterol lowering medication in use | INR | 0,246 | 0,498 | 0,494 | 0,621 | 0,482 | 3,396 | 1,279 |
| Psychotropic medication in use | INR | 0,574 | 0,593 | 0,968 | 0,333 | 0,556 | 5,669 | 1,775 |
| Vitamin D medication in use | INR | -0,419 | 0,437 | -0,958 | 0,338 | 0,279 | 1,549 | 0,658 |
| Anxiety disorder | INR | -15,38 | 2099 | -0,007 | 0,994 | 0,000 | INF | 0,000 |
| Depression | INR | -0,697 | 0,762 | -0,915 | 0,360 | 0,112 | 2,218 | 0,498 |
| Appendectomy | INR | 0,017 | 0,782 | 0,022 | 0,983 | 0,220 | 4,708 | 1,017 |
| Splenectomy | INR | 0,000 | 73977 | 0,000 | 1,000 | 0,000 | INF | 1,000 |
| Tonsillectomy | INR | -0,377 | 0,451 | -0,835 | 0,404 | 0,283 | 1,662 | 0,686 |
| Epilepsy | INR | -16,92 | 5695 | -0,003 | 0,998 | 0,000 | INF | 0,000 |
| Parkinsons disease | INR | 5,047 | 2,038 | 2,476 | 0,013 | 2,865 | 8441 | 156 |
| Angina pectoris | INR | 2,258 | 1,464 | 1,542 | 0,123 | 0,542 | 169 | 9,563 |
| Arrythmia | INR | -16,33 | 3334 | -0,005 | 0,996 | 0,000 | INF | 0,000 |
| Hypertension | INR | -0,045 | 0,505 | -0,088 | 0,930 | 0,355 | 2,574 | 0,956 |
| Myocardial infarction | INR | -15,69 | 1232 | -0,013 | 0,990 | 0,000 | INF | 0,000 |
| Peripheral arterial disease | INR | 1,523 | 1,234 | 1,235 | 0,217 | 0,409 | 51 | 4,587 |
| Stroke | INR | -16,37 | 2070 | -0,008 | 0,994 | 0,000 | INF | 0,000 |
| Venous thromboembolism | INR | 0,402 | 1,106 | 0,363 | 0,716 | 0,171 | 13 | 1,495 |
| Diabetes type 1 | INR | -16,16 | 5608 | -0,003 | 0,998 | 0,000 | INF | 0,000 |
| Diabetes type 2 | INR | -0,125 | 1,081 | -0,116 | 0,908 | 0,106 | 7,346 | 0,883 |
| Hypercholesterolemia | INR | 0,187 | 0,496 | 0,377 | 0,706 | 0,456 | 3,183 | 1,205 |
| Hyperthyroidism | INR | -16,16 | 5608 | -0,003 | 0,998 | 0,000 | INF | 0,000 |
| Hypothyreoidism | INR | -14,91 | 2050 | -0,007 | 0,994 | 0,000 | INF | 0,000 |
| Inflammatory bowel disease | INR | -15,72 | 7881 | -0,002 | 0,998 | 0,000 | INF | 0,000 |
| Inflammatory arthritis | INR | -16,75 | 3239 | -0,005 | 0,996 | 0,000 | INF | 0,000 |
| Osteoarthritis | INR | 0,529 | 1,117 | 0,474 | 0,636 | 0,190 | 15 | 1,698 |
| Osteoporosis | INR | -16,05 | 2004 | -0,008 | 0,994 | 0,000 | INF | 0,000 |
| Asthma | INR | -0,665 | 1,054 | -0,631 | 0,528 | 0,065 | 4,059 | 0,514 |
| COPD | INR | 0,389 | 1,108 | 0,351 | 0,725 | 0,168 | 13 | 1,476 |
| High grade squamous intraepithelial anal lesions | INR | -18,97 | 6523 | -0,003 | 0,998 | 0,000 | INF | 0,000 |

Supplementary table 2G: Antiretroviral regimen in use as predictive of INR phenotype compared to IR phenotype in the discovery cohort. Multivariate logistic regression analysis with sex and age as covariates.

| name | outcome | beta | se | z value | Pr(>\|z\|) | 2.5 % | 97.5 % | OR |
| --- | --- | --- | --- | --- | --- | --- | --- | --- |
| INSTI in use | INR | -0,3943258 | 0,26486956 | -1,48875469 | 0,13655198 | 0,40113365 | 1,13293204 | 0,67413439 |
| NNRTI in use | INR | 0,17603891 | 0,26216211 | 0,67148876 | 0,50190922 | 0,71334551 | 1,99345079 | 1,19248446 |
| Protease in use | INR | 0,47222627 | 0,37479432 | 1,25996112 | 0,20768339 | 0,76923628 | 3,34280285 | 1,60356018 |
| 3DR-INSTI | INR | -0,16091348 | 0,27976939 | -0,57516471 | 0,56517991 | 0,49201237 | 1,47318165 | 0,85136573 |
| 2DR-INSTI | INR | -0,65707409 | 0,47409034 | -1,38596811 | 0,16575664 | 0,20468684 | 1,31275227 | 0,51836581 |
| 3DR-NNRTI | INR | 0,23540092 | 0,26224801 | 0,89762711 | 0,36938438 | 0,75684581 | 2,11572508 | 1,265416 |
| 3DR-Protease | INR | 0,2007043 | 0,53331735 | 0,37633184 | 0,70667021 | 0,42973846 | 3,47636462 | 1,2222633 |

Supplementary table 2H: Antiretroviral regimen in use as predictive of INR phenotype compared to IR phenotype in the validation cohort. Multivariate logistic regression analysis with sex and age as

covariates.

| name | outcome | beta | se | z value | Pr(>\|z\|) | 2.5 % | 97.5 % | OR |
| --- | --- | --- | --- | --- | --- | --- | --- | --- |
| INSTI in use | INR | 0,25609503 | 0,46596784 | 0,54959807 | 0,58259508 | 0,51830824 | 3,21998022 | 1,29187549 |
| NNRTI in use | INR | -0,33803395 | 0,48948995 | -0,69058406 | 0,48982697 | 0,27323676 | 1,86143689 | 0,71317107 |
| Protease in use | INR | 1,14828952 | 0,57055219 | 2,01259333 | 0,04415743 | 1,03048317 | 9,64607658 | 3,15279551 |
| 3DR-INSTI | INR | 0,10050276 | 0,42345433 | 0,23734026 | 0,81239283 | 0,48217292 | 2,53567024 | 1,1057267 |
| 2DR-INSTI | INR | 0,00966736 | 0,65619872 | 0,01473237 | 0,9882457 | 0,27902371 | 3,6538932 | 1,00971424 |
| 3DR-NNRTI | INR | -0,24437314 | 0,49011795 | -0,49860067 | 0,61806073 | 0,29969599 | 2,04672385 | 0,78319534 |
| 3DR-Protease | INR | 0,90591462 | 0,69663338 | 1,30041806 | 0,19345772 | 0,63162355 | 9,6919045 | 2,47419384 |

Abbreviations: Abbreviations: BMI; body mass index, cART: combination antiretroviral therapy; COPD: chronic obstructive pulmonary disease; CVD: cardiovascular disease; IV: intravenous; MSM: man having sex with man; OR: odds ratio; INSTI: integrase strand transfer inhibitor; NNRTI: non-nucleoside reverse transcriptase inhibitor; protease: protease inhibitor; 3DR-INSTI: three antiretroviral drug regimens with an integrase strand transfer inhibitor in use; 2DR-INSTI: two antiretroviral drug regimens with an integrase strand transfer inhibitor in use; 3DR-NNRTI: three antiretroviral drug regimens with an non-nucleoside reverse transcriptase inhibitor in use; 3DR-Protease: three antiretroviral drug regimens with an protease inhibitor in use;

**Supplementary table 3:** Flow cytometry results comparing INR to IR. Linear models using sex, age, seasonality and COVID-19 vaccination as covariates.

Supplementary table 3A: Flow cytometry absolute counts comparing INR to IR in discovery cohort.
Table is provided in a separate excel file

Supplementary table 3B: Flow cytometry absolute counts comparing INR to IR in the validation cohort.

Table is provided in a separate excel file

Supplementary table 3C: Flow cytometry percentages comparing INR to IR in the validation cohort.

Table is provided in a separate excel file

Supplementary table 3D: Flow cytometry percentages comparing INR to IR in the validation cohort.

Table is provided in a separate excel file

Supplementary table 3E: Flow cytometry MFI comparing INR to IR in the validation cohort.

Table is provided in a separate excel file

Supplementary table 3F: Flow cytometry MFI comparing INR to IR in the validation cohort.

Table is provided in a separate excel file

**Supplementary table 4:** FDR (discovery) and nominal p-values (validation) of peripheral blood mononuclear cell cytokine production analysis comparing immunological non-responders to immunological responders. Non-parametric rank based model using sex, age and lymphocyte/monocyte ratio as covariates.
Supplementary table 4A: FDR p-values of the cytokine production after 24 hours of stimulation with various stimuli in the discovery cohort.

|  | **IL1RA** | **IL10** | **IL6** | **IL8** | **IL1b** | **TNF** | **MCP1** | **MIP1a** |
| --- | --- | --- | --- | --- | --- | --- | --- | --- |
| **CMV** | 1,000 |  | 1,000 | 1,000 | 0,991 | 0,569 | 0,057 | 0,569 |
| **Spneu** | 0,876 | 1,000 | 0,601 | 0,453 | 1,000 | 1,000 | 0,569 | 1,000 |
| **LPS** | 0,972 | 1,000 | 0,465 | 0,038 | 1,000 | 1,000 | 0,687 | 1,000 |
| **IMQ** | 1,000 |  | 0,569 | 1,000 | 0,687 |  | 1,000 | 1,000 |
| **HIVENV** | 1,000 |  | 1,000 | 1,000 | 0,569 | 1,000 | 1,000 | 1,000 |
| **PolyIC** | 1,000 |  |  |  |  |  | 1,000 | 1,000 |
| **IL1a** | 1,000 |  | 1,000 | 0,687 |  |  | 0,687 | 1,000 |

Supplementary table 4B: Nominal p-values of the cytokine production after 24 hours of stimulation with various stimuli in the validation cohort.

|  | **IL1RA** | **IL10** | **IL6** | **IL8** | **IL1b** | **TNF** | **MCP1** | **MIP1a** |
| --- | --- | --- | --- | --- | --- | --- | --- | --- |
| **CMV** | 0,362 |  | 1,000 | 0,854 | 0,409 | 1,000 | 0,615 | 0,678 |
| **Spneu** | 0,891 | 0,202 | 0,378 | 0,850 | 0,723 | 0,801 | 0,574 | 0,076 |
| **LPS** | 0,336 | 0,219 | 0,611 | 0,308 | 0,160 | 0,541 | 0,661 | 0,045 |
| **IMQ** | 0,744 |  | 0,068 | 0,381 | 0,129 |  | 0,677 | 0,003 |
| **HIVENV** | 0,914 |  | 1,000 | 0,630 | 0,104 | 1,000 | 0,054 | 1,000 |
| **PolyIC** | 0,591 |  |  |  |  |  | 0,399 | 1,000 |
| **IL1a** | 0,837 |  | 1,000 | 0,142 |  |  | 0,213 | 1,000 |

Supplementary table 4C: FDR p-values of the cytokine production after 7 days of stimulation with various stimuli in the discovery cohort.

|  | **IL22** | **IL5** | **IL10** | **IFNy** | **IL17** |
| --- | --- | --- | --- | --- | --- |
| **C.alb.con** | 0,007 | 0,920 | 0,353 | 0,732 | 0,660 |
| **E.coli** | 0,001 |  | 0,289 | 0,003 | 1,000 |
| **MTB** | 0,021 | 1,000 | 0,137 | 0,054 | 1,000 |
| **PHA** | 0,004 | 0,042 | <0,001 | 0,279 | 0,009 |
| **S.aureus** | 0,006 |  | 0,389 | 0,013 | 0,215 |
| **S.pneu** | 0,001 | 0,353 | 0,375 | <0,001 | 0,007 |
| **C.alb.hy** | 0,006 | 0,175 | 0,220 | 0,251 | 0,294 |

Supplementary table 4D: Nominal P-values of the cytokine production after 7 days of stimulation with various stimuli in the validation cohort.

|  | **IL22** | **IL5** | **IL10** | **IFNy** | **IL17** |
| --- | --- | --- | --- | --- | --- |
| **C.alb.con** | 0,155 | 0,066 | 0,298 | 0,969 | 0,424 |
| **E.coli** | 0,396 |  | 0,415 | 0,040 | 1,000 |
| **MTB** | 0,822 | 1,000 | 0,172 | 0,600 | 1,000 |
| **PHA** | 0,019 | 0,969 | <0,001 | 0,947 | 0,019 |
| **S.aureus** | 0,158 |  | 0,739 | 0,528 | 0,038 |
| **S.pneu** | 0,006 | 0,259 | 0,190 | 0,046 | 0,106 |
| **C.alb.hy** | 0,004 | 0,156 | 0,252 | 0,010 | 0,005 |

Abbreviations: C.alb.con: *Candida albicans* conidia; C.alb.hy: *Candida albicans* hyphae; CMV: cytomegalovirus protein; E. coli: *Escherichia coli;* FDR: false discovery rate, HIVENV: HIV envelope protein; IL: interleukin; IMQ: imiquimod; LPS: lipopolysaccharide; MCP1: monocyte chemoattractant protein-1; MIP-1α: macrophage inflammatory proteins 1α; MTB: *Mycobacterium tuberculosis;* PBMC: peripheral blood mononuclear cells; PHA: phytohemagglutinin; PolyIC: Polyinosinic:polycytidylic acid; S.aureus: *Staphylococcus aureus;* Spneu: *Streptococcus pneumoniae*; TNF: tumor necrosis factor
